# Nuclear BK channels regulate CREB phosphorylation in RAW264.7 macrophages

**DOI:** 10.1007/s43440-021-00229-z

**Published:** 2021-03-13

**Authors:** Anna Selezneva, Minae Yoshida, Alasdair Gibb, Dean Willis

**Affiliations:** Department of Neuroscience, Physiology and Pharmacology, University College London, Gower Street, London, WC1E 6BT UK

**Keywords:** Macrophage, Nuclear, BK channel, CREB phosphorylation, CaMKII, CaMKIV

## Abstract

**Background:**

Macrophages are important cells of the innate immune system and contribute to a variety of physiological and pathophysiological responses. Monovalent and divalent ion channels have been studied in macrophage function, and while much research is still required, a role for these channels is beginning to emerge in macrophages. In addition to the plasma membrane, ion channels are also found in intracellular membranes including mitochondrial, lysosomal and nuclear membranes. While studying the function of plasma membrane located large conductance voltage- and calcium-activated potassium channels (BK channels) in a macrophage cell line RAW264.7, we became aware of the expression of these ion channels in other cellular locations.

**Methods:**

Immunofluorescence and Western blot analysis were used to identify the expression of BK channels. To demonstrate a functional role for the nuclear located channel, we investigated the effect of the lipid soluble BK channel inhibitor paxilline on CREB phosphorylation.

**Results:**

Treatment of resting macrophages with paxilline resulted in increased CREB phosphorylation. To confirm a role for nuclear BK channels, these experiments were repeated in isolated nuclei and similar results were found. Ca^2+^ and calmodulin-dependent kinases (CaMK) have been demonstrated to regulate CREB phosphorylation. Inhibition of CaMKII and CaMKIV resulted in the reversal of paxilline-induced CREB phosphorylation.

**Conclusions:**

These results suggest that nuclear BK channels regulate CREB phosphorylation in macrophages. Nuclear located ion channels may therefore be part of novel signalling pathways in macrophages and should be taken into account when studying the role of ion channels in these and other cells.

**Graphic abstract:**

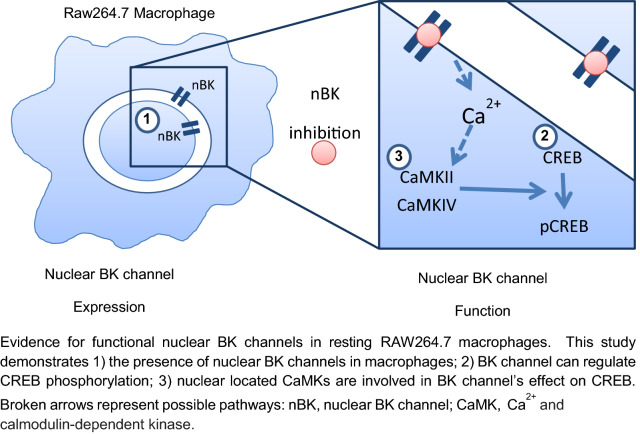

**Supplementary Information:**

The online version contains supplementary material available at 10.1007/s43440-021-00229-z.

## Introduction

Large conductance voltage- and Ca^2+^-activated potassium channels (BK channels) are characterized by a large single channel conductance (~ 100–300pS) and high sensitivity to intracellular Ca^2+^ concentration [1]. The channel itself is made up of four channel-forming α-subunits which are the product of the KCNMA1 gene otherwise known as Slo1 [1–3]. In addition to the pore-gate domain, each α-subunit also contains a voltage sensing domain and Ca^2+^ and Mg^2+^ binding domain. These α-subunit domains are responsible for the channel opening in response to a rise in intracellular Ca^2+^ and/or membrane voltage [3–5]. A number of ligands have been demonstrated to modulate channel opening, these include carbon monoxide, heme and various lipids [6–8]. While four α-subunit configuration is the minimal stoichiometry for an active channel, the binding of various combinations of auxiliary subunits, either β1-4 and/or γ1-4, modify the channel’s opening properties [9].

As a result of their large conductance, opening of BK channels allows the rapid movement of K^+^ across lipid membranes which causes a decrease in membrane potential and hyperpolarizes the membrane [1, 3, 5]. At the cellular level, the BK channels regulate membrane excitability, ion homeostasis, calcium signalling and cell volume and have been implicated in numerous areas of physiology, such as hearing, neuronal activity, vascular relaxation, circadian rhythms and cryoprotection in the heart [10–14]. In pathophysiological situations, BK channels have been linked to epilepsy, chronic pain and cancer [15, 16].

To date, the majority of research carried out on BK channels concerns the cellular function of plasma membrane located BK channels. However, BK channels are also located on intracellular organelle membranes such as mitochondria, lysosomes and nuclei [17–19]. While BK channels have been detected in the nuclear membranes of a variety of cells including endothelial cells, neurons and microglial cells, their role in intracellular signalling pathways is incompletely understood [10, 19, 20]. Proposed functions for nuclear membrane BK channels include the regulation of ion concentration in the perinuclear space, nuclear calcium signalling and the regulation of transcription factor activity [19, 21].

Macrophages are cells of the innate immune system and are important in the initiation, propagation and resolution of inflammatory responses, the killing of invading microbes, the removal of noxious material and initiation of tissue repair. Under physiological conditions, macrophages contribute to tissue homeostasis by removing the extracellular debris which accumulates during normal physiological processes and act as early warning monitors of changes within tissue environment. An important ability of macrophages is to monitor their local environment and activate an appropriate response. To accomplish this, macrophages express a large number of different Pattern Recognition Receptors (PRRs). Toll-like receptor-4 (TLR-4) is the archetypal PRR which binds and is activated by lipopolysaccharide (LPS). This results in activation of the transcription factor Nuclear factor-kappa B (NF-κB) with the resultant production of a large number of pro-inflammatory mediators, such as Tumour Necrosis Factor-alpha (TNFα) and inducible cyclooxygenase-2 (COX-2) which produces prostaglandins, various proteases and reactive oxygen species. It is important to note that macrophages are highly heterogentic in nature and show distinct anatomical and biological features depending on the tissue they are located in and how they are activated. Underlying this heterogeneity is a complex series of interconnected, feedback rich, signalling cascades which regulate the macrophage response. While these cascades have been extensively researched, the role of ion channels, particularly intracellular ion channels, has received little attention in these cascades [22, 23].

cAMP response element binding protein (CREB) is a transcription factor involved in cell survival, proliferation, cell adaptation and differentiation. CREB plays a major role in the immune system where it regulates the expression of a variety of inflammatory mediators in leukocytes and macrophages [24–27]. The transcriptional regulatory properties of CREB are dependent on its phosphorylation at serine 133 and this is carried out by a number of Ca^2+^ and calmodulin-dependent protein kinases (CaMKs) [28].

In this study, we add to knowledge on BK channels and macrophage signalling cascades by showing the presence of nuclear BK channels in murine macrophages and demonstrating that they regulate CREB phosphorylation.

## Materials and methods

### Materials

All cell culture reagents were obtained from Thermo Fisher Scientific, US unless otherwise stated. All chemicals were obtained from Sigma-Aldrich, UK unless otherwise stated. Ultrapure LPS (Invivogen, France), paxilline (Cayman Chemical Co., US), TATCN21 (sequence KRPPKLGQIGRSKRVVIEDDR) and scrambled control (sequence VKEPRIDGKPVRLRGQKSDRI) were obtained from Genscript (NJ, USA) [29]. Mouse monoclonal anti-mouse BKα antibody, clone L6/60 (EMD Millipore, US), Rabbit polyclonal anti-mouse lamin B1 antibody (Abcam plc., US), Rabbit monoclonal anti-mouse CREB antibody and Rabbit monoclonal anti-mouse Phospho (Ser133)-CREB antibody (Cell Signaling Technology, US), Mouse monoclonal anti-human COX IV antibody (Abcam plc. US), Rabbit monoclonal anti-human GADPH antibody (Cell Signalling Technology, US), Goat polyclonal anti-human Calnexin antibody (Sicgen, Portugal). All primary antibodies had been reported to detect the equivalent protein in mouse. Goat anti-rabbit IgG HRP-linked secondary antibody (Cell Signalling Technology, US), Rabbit anti-mouse IgG HRP-linked secondary antibody (Abcam plc., US), Rabbit anti-Goat IgG HRP-linked secondary antibody (R and D systems, US), F(ab)’ fragment affinity-purified unconjugated goat anti-mouse IgG (Thermo Fisher Scientific, US).

### Cell culture

RAW264.7 murine macrophage cell line, European Collection of Cell Culture, were cultured in DMEM containing 10% FBS, 100 unit/ml penicillin and 100 ug/ml streptomycin at 37 °C, 5% CO_2_. The passage number never exceeded 16.

### Nuclei and nuclear membrane preparation

1 × 10^7^ of RAW264.7 macrophages were resuspended in nuclei buffer (8 mM Tris–HCl, 2 mM Tris-base, 10 mM NaCl, 3 mM MgCl_2_, pH 7.5) containing 0.8% NP-40 and centrifuged at 180 g for 15 min at 4 °C, and the pellet containing nuclei was collected. This was repeated twice and then followed by two washes in nuclei buffer only. Nuclear integrity was checked using trypan blue and light microscopy [30].

For nuclear membrane preparation, isolated nuclei were resuspended in Triton buffer (300 mM sucrose, 100 mM NaCl, 3 mM MgCl_2_, 0.5 mM CaCl_2_, 1 mM DTT, 1% Triton X100) for 7 min at RT. The resultant nuclear membrane supernatant and denuded nuclei were separated by centrifugation at 650 g for 5 min at RT. Nuclei preparations were used immediately for nuclei activation studies then stored at − 80 °C prior to analysis [31].

### Cell and nuclei activation

1 × 10^7^ cells or nuclei were used for each treatment. Isolated nuclei were placed in an EGTA-buffered intracellular medium (125 mM KCl, 2 mM K_2_HPO_4_, 40 mM HEPES, 0.1 mM MgCl_2_, 10 mM EGTA, pH 7.2) and incubated with increasing concentrations, 0–200 nM, of Ca^2+^ or with 100 nM paxilline for 5 min at 37 °C. Whole cells were activated by the addition of 100 ng/ml LPS or 10–1000 nM paxilline for 25 min at 37 °C. For kinase inhibitor studies, whole cells were first pre-treated with 5 M STO-609, 5μM TATCN21or relevant control for 10 min (isolated nuclei for 5 min) prior to paxilline treatment. Cells or nuclei were collected after the experiment and stored at − 80 °C prior to analysis.

### Western blot analysis

Samples were lysed by sonication at 4 °C in PBS containing protease inhibitor cocktail. The protein concentration in each sample was measured by Bradford assay, adjusted to 1 mg/ml in sample loading buffer, and 10–20 ug of protein was loaded in to each well. For the analysis of BKα expression, samples were also prepared and put in loading buffer on a cell number basis, 100,000 cells equivalent per well. Samples were run on Mini-PROTEAN system (Bio-Rad) with 12% polyacrylamide gels used for the analysis of CREB and pCREB and 7.5% gels for the analysis BKα and lamin B1, and 4–20% gradient gels for organelle markers.

Separated proteins were transferred to nitrocellulose membranes. Membranes were blocked with 5% BSA in TBST (0.1% Tween 20 in TBS: Tris-Buffered Saline, 10 mM Tris, pH 7.4) overnight at 4 °C. 1.25 g/ml F(ab)’ fragment affinity-purified unconjugated goat anti-mouse IgG was added to the blocking solution when using mouse monoclonal primary antibodies. The membranes were incubated with primary antibodies overnight at room temperature followed by appropriate secondary antibodies for 2 h at room temperature. Protein bands were visualised by chemiluminescence using Pierce chemiluminescent Western Blotting Substrate (Thermo Fisher Scientific, US.) and scanned by Typhoon 9410 Variable Mode Imager (GE Healthcare). Band intensity for pCREB and CREB bands was analysed using ImageJ Software. The pCREB/CREB ratio was calculated for each sample. For statistical analysis of CREB phosphorylation, data from individual experiments were normalised using feature scaling.

### Immunofluorescence staining

Cells were plated onto glass circular coverslips, 1.1 × 10^4^ cells per slide. 24 h later, cells were fixed with 4% paraformaldehyde and incubated for 1 h with a block solution (1.25 g/ml F(ab)’ fragment affinity-purified unconjugated goat anti-mouse IgG, 10% goat serum and 4% bovine serum albumin (BSA) in PBS) followed by 5 min incubation with 0.01% Triton X100. The coverslips were then incubated with anti-BKα antibody diluted in 5% goat serum and 2% BSA in PBS at 4 °C overnight, followed by secondary antibody, goat anti-mouse IgG (H and L) Alexa Fluor 488 conjugate for 2 h, and DAPI. Coverslips were mounted on glass slides with Vectashield (Vector Laboratories, US). Fluorescence was visualized using a confocal microscopy (Zeiss LSM510 Meta, Carl Zeiss, UK). Experiments were repeated at least 3 times and figures show representative images.

### TNFα measurements

1 × 10^5^ of RAW264.7 macrophages were plated into 24 well plates and stimulated with indicated drug. 4 h later, the culture medium was collected and stored at − 80 °C prior to analysis. TNFα concentration in the medium was assessed by ELISA (BD Biosciences, US).

### Statistical analysis

To determine the number of experimental repeats required for statistical analysis, statistical power calculation was computed in G*Power 3.1.9.3 Software. The number of repeats used is quoted in each results section. A Shapiro–Wilk test indicated that the data collected were not normally distributed, except for the data reported in Fig. 4b. To aid clarity, we will report non-parametric methods and metrics in all the figures, a full statistical report of the results can be found in the supplementary material. Kruskal–Wallis one-way analysis of variance was used to test for statistical significance among treatment groups, followed by Dunn’s post hoc test. The tests were performed using Graphpad Prism 8.

## Results

**Fig. 1:**
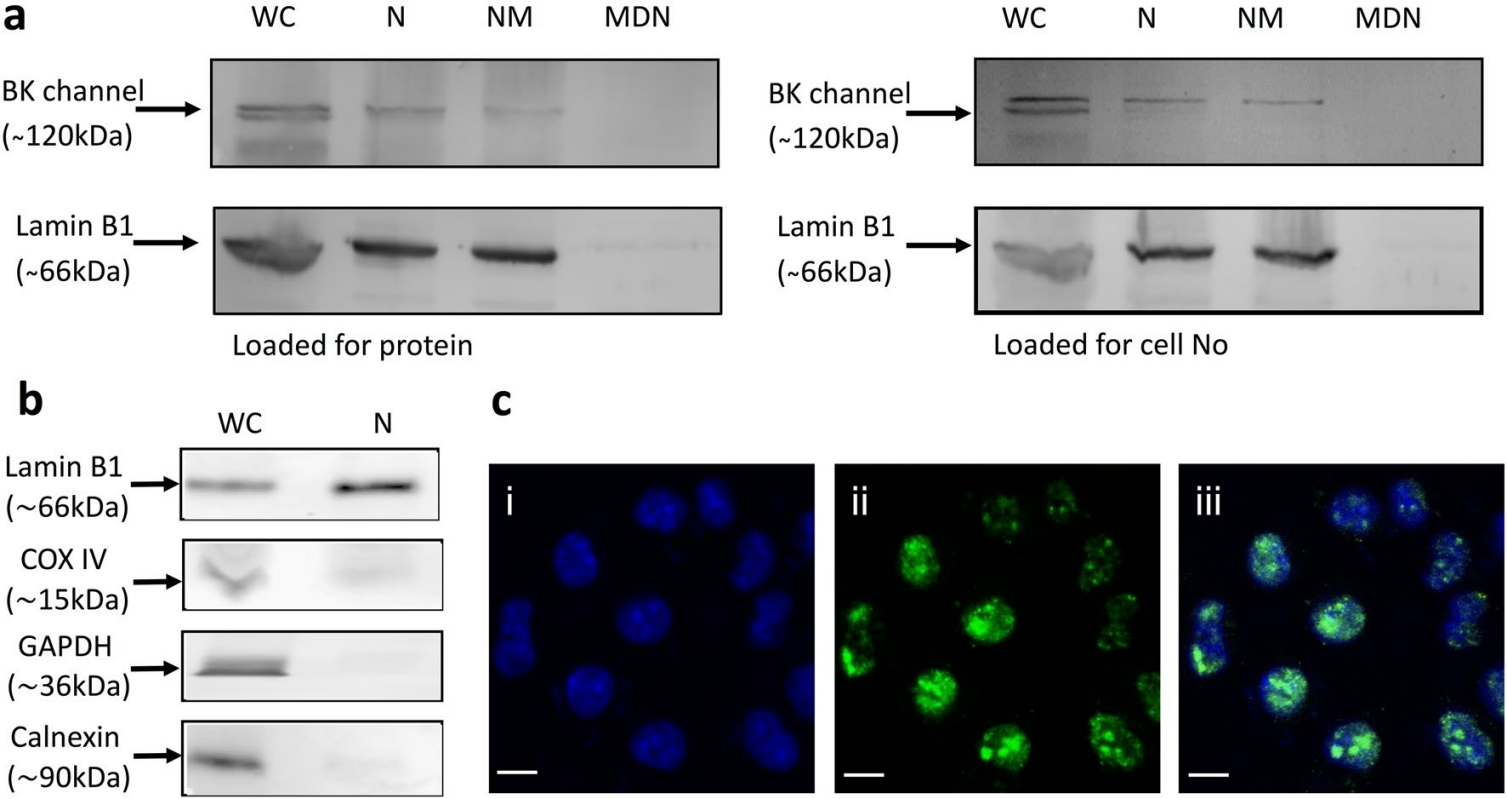
Expression of BKα in RAW264.7 macrophages. **a** Representative Western blots of BK channel and lamin B1 expression in RAW 264.7 macrophages; whole cell (WC); nuclei (N); nuclei membrane (NM); membrane denuded nuclei preparations (MDN) preparations. Gels loaded for protein 20ug or initial cell number 100,000 equivalents. **b** Representative blots of lamin B1, COX IV, GAPDH and calnexin in whole cell and nuclei preparations **c** Representative immunofluorescence of whole cells for BK channel. (i) DAPI only; (ii) BK channel antibody; (iii) Merged DAPI + BK channel antibody. Scale bar 5μM

Western blot analysis was used to determine if RAW264.7 murine macrophages express BK channel α-subunits in their nuclei. Lamin B1 was used to confirm the isolation of the nucleus and nuclear membrane. As expected, removing the nuclear membrane resulted in decreased lamin B1 expression in denuded nuclei. A 120 kDa protein band which corresponds to the BK α-subunit was found in all preparations except the denuded nuclear lysate (Fig. 1a). This result clearly demonstrates the presence of BK α-subunit in the nuclear membrane in resting RAW264.7 macrophages. It was also noted that Western blot analysis of whole cell lysates resulted in the expression of a protein band doublet for the BK channel α-subunit while in nuclear preparations, the α-subunit was seen as a single protein band (Fig. 1a). To exclude the possibility that BK channel expression in the nuclear lysates was due to contamination, lysates were analysed for cytochrome c oxidase subunit IV (COX IV), a mitochondrial marker; GAPDH, a cytoplasmic marker; and calnexin, an endoplasmic reticulum membrane marker. The minimum staining of these markers in the nuclear lysates demonstrates that it is highly unlikely that contamination contributes significantly to the BK channel staining seen in the nuclear preparations (Fig. 1b). Immunolocalization studies of intact whole cells confirmed the Western blot results with positive staining for BK α-subunit being found in the nuclei of approximately 90% of resting macrophages (Fig. 1c). It was noted that staining for BK channel was diffuse in the macrophage nuclei and would suggest that BK channel, or its variants, were not only found in the nuclear membrane as indicated from the western blot analysis, but may also be present within the nucleus. Finally, we noted that immunofluorescence staining appeared not to indicate BK channel expression on the plasma membrane. This is in line with electrophysiological and plasma membrane protein expression experiments in our lab which demonstrated that resting RAW264.7 macrophages have limited plasma membrane BK channel expression compared to cells which are activated for 12–24 h with LPS (manuscript in preparation).

Reports have demonstrated a role for the BK channel in the regulation of CREB phosphorylation in neurons [19]. In this report, BK channel opening appears to inhibit CREB phosphorylation by controlling the perinuclear concentration of Ca^2+^. As CREB is reported to have a role in regulating macrophage function [24–26], we investigated if blocking the nuclear BK channel could affect CREB phosphorylation in these cells.

**Fig. 2:**
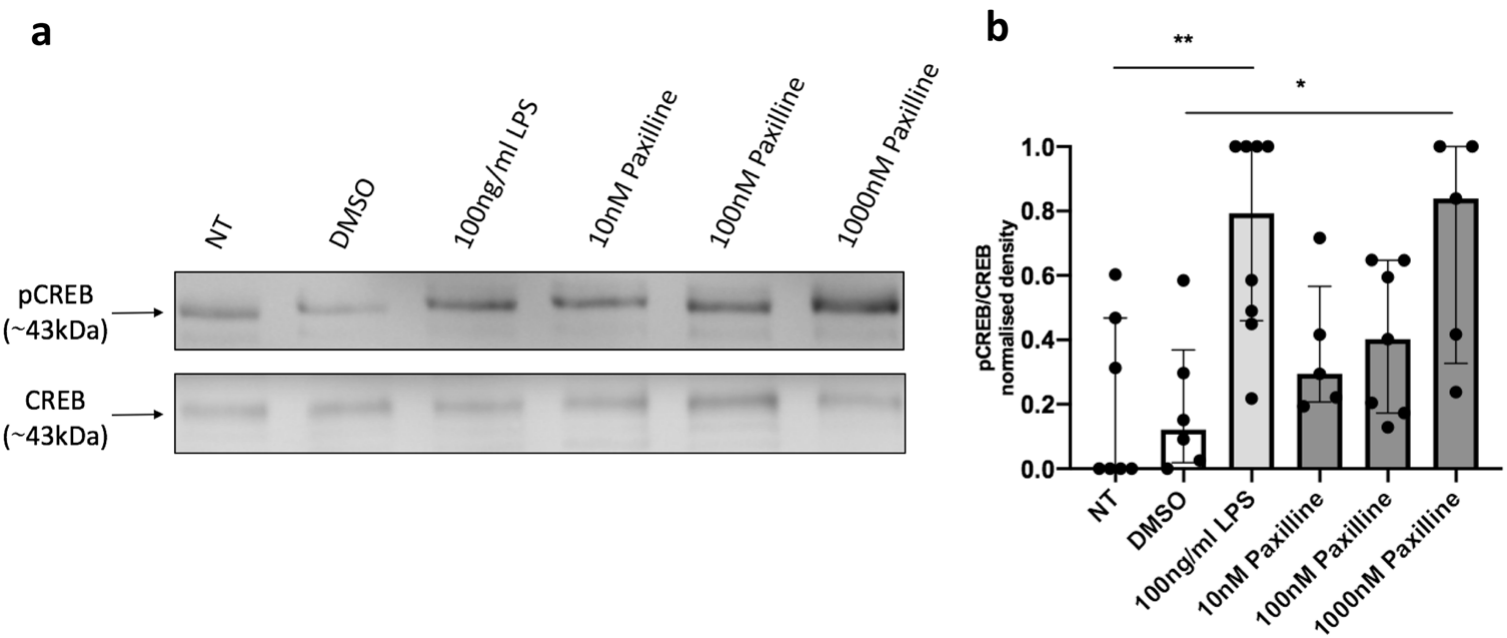
Blockade of BK channels induces CREB phosphorylation. Western blot of pCREB and CREB expression in whole cells. Cells were incubated for 25 min with indicated solutions. **a** Representative Western blot from one experiment. 10 ug protein was loaded into each lane. **b** Densitometry analysis of immunoblots. The results are 5–8 independent experiments. Kruskal–Wallis one-way analysis of variance, followed by Dunn’s post hoc test. Error bars represent median and interquartile ranges. *p = 0.0132 was, **p = 0.0034. *NT* no treatment control, *DMSO* 0.01% DMSO

The TLR4 ligand, LPS, is the archetypal macrophage activating agent. Dose response curves established that 100 ng/ml LPS resulted in maximal RAW264.7 macrophage activation, as measured by TNFα released 4 h after stimulation (supplementary Figure 1a). 100 ng/ml LPS treatment for 25 min caused a significant increase (**p = 0.0034) in CREB phosphorylation, (Fig. 2).

Paxilline is a lipid soluble selective BK channel blocker and is therefore able to bind and inhibit intracellular BK channels. Paxilline treatment of RAW264.7 macrophages resulted in a dose-dependent increase in CREB phosphorylation with 1 μM of the blocker causing an approximately eightfold increase in pCREB expression (*p = 0.0132) compared to DMSO control (Fig. 2). It is highly unlikely that this increase in CREB phosphorylation was due to the BK channel inhibitor causing a classical activation of the macrophage as TNFα release could not be detected 4 h after 1 μM paxilline treatment (supplementary data Figure 1b).

**Fig. 3:**
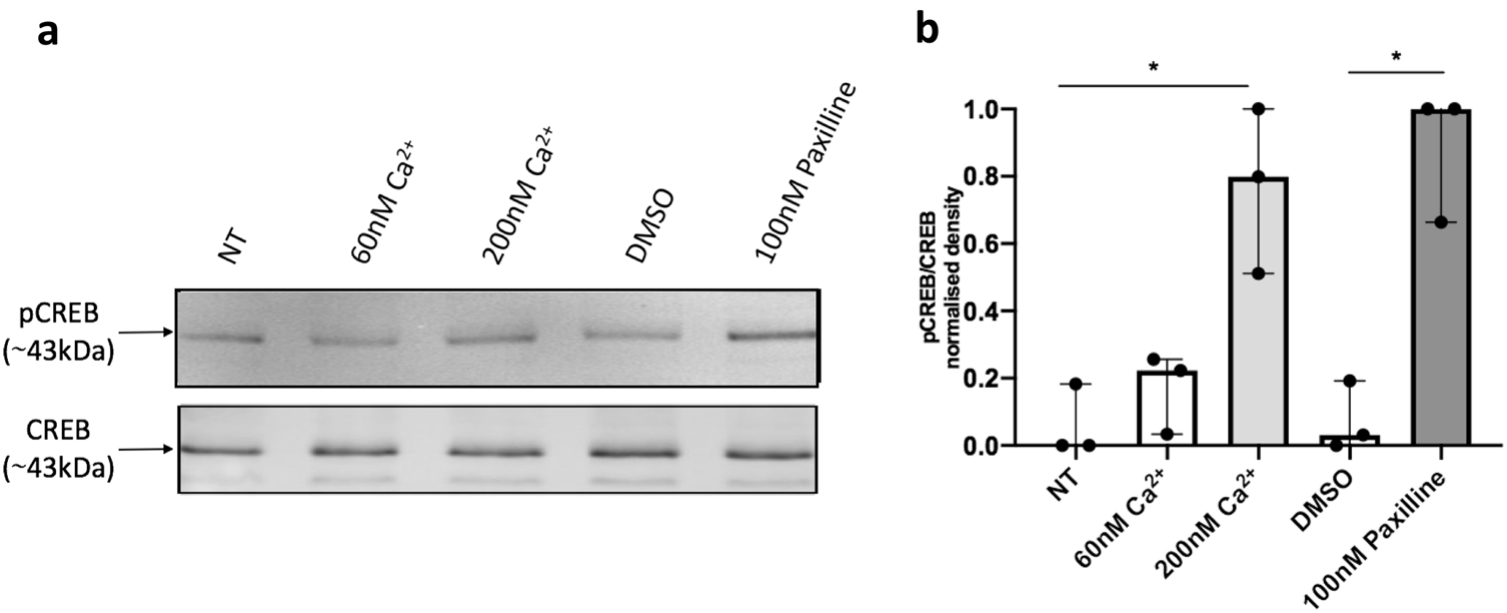
Blockade of nuclear BK channels induces CREB phosphorylation in isolated nuclei. Western blot of pCREB and CREB expression in nuclei, stimulated with indicated solution for 5 min. **a** Representative Western blot from one experiment. 10ug protein was loaded into each lane. **b** Densitometry analysis of immunoblots. The results are 3 independent experiments. Kruskal–Wallis one-way analysis of variance, followed by Dunn’s post hoc test. Error bars represent median and interquartile ranges. NT vs. 200 nM Ca^2+^ *p = 0.0168. DMSO vs. 100 nM paxilline *p = 0.0168. *NT* no treatment control, *DMSO* 0.01% DMSO

To demonstrate that paxilline’s effect on CREB phosphorylation was most likely mediated by nuclear BK channels, and not BK channels at other cellular locations, we investigated the effect of paxilline on CREB phosphorylation in isolated RAW264.7 macrophage nuclei. 100 nM paxilline caused a significant increase in pCREB levels in the nucleus (*p = 0.0168). This result was comparable to the increase in pCREB caused by the treatment of the nuclei with high concentrations, 200 nM, of Ca^2+^ (*p = 0.0168) which is known to cause CREB phosphorylation (Fig. 3) [3, 5, 19]. Taken together with the results in Fig. 2, the data demonstrate that the block of nuclear BK channels results in CREB phosphorylation.

We hypothesised that the block of nuclear BK channels results in an increase in nuclear Ca^2+^ concentrations which would activate Ca^2+^ dependent kinases and lead to CREB phosphorylation. Two Ca^2+^ dependent kinases which have been found in the nucleus and have been implicated in CREB phosphorylation in neurons are Ca^2+^ and calmodulin-dependent kinase type-II (CaMKII) and Ca^2+^ and calmodulin-dependent kinase type-IV (CaMKIV) [19, 32]. To investigate the involvement of these Ca^2+^ dependent kinases in the increase in CREB phosphorylation seen after BK channel inhibition, STO-609, which selectively inhibits Ca^2+^ and calmodulin-dependent kinase kinase 2 (CaMKK2) which is upstream of CaMKIV [32] and the CaMKII peptide inhibitor, tatCN21 [29], were added to our system.

**Fig. 4:**
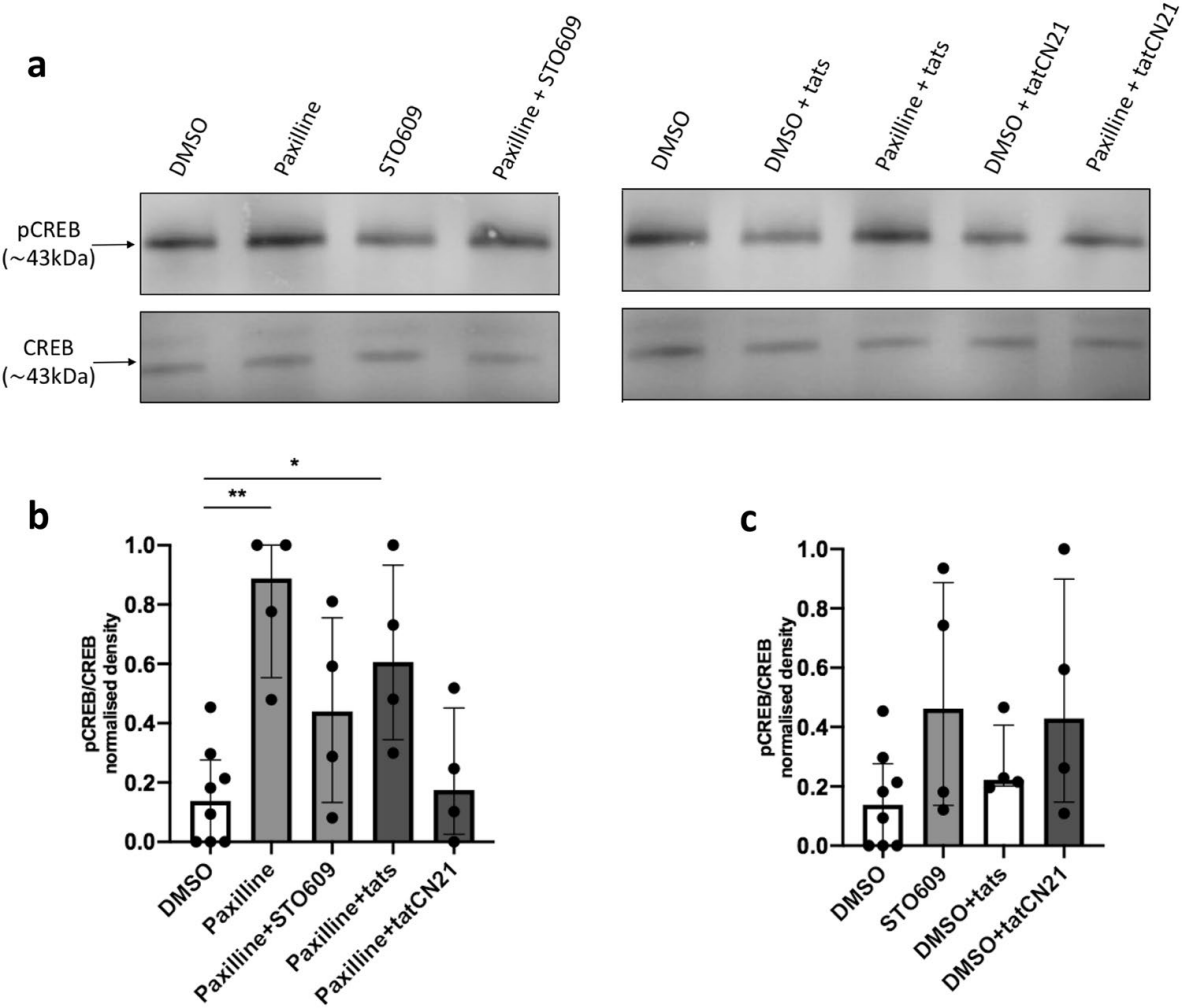
Ca^2+^ and calmodulin-dependent kinase inhibitors reduce paxilline-induced CREB phosphorylation in whole cells. Western blot of pCREB and CREB expression following pre-treatment of whole cells with 5 μM STO609, 5 μM tatCN21, 5 μM tats or 100 mM NaOH for 10 min and subsequent stimulation with 1 μM paxilline or DMSO for 25 min. **a** Data representative of one repeat. Approximate molecular weight of the protein of interest is indicated in kDa. 10 ug protein was loaded into each lane. **b**, **c** Densitometry analysis of immunoblots. The results are representative of at least 4 independent experiments. Kruskal–Wallis one-way analysis of variance, followed by Dunn’s post hoc test. Error bars represent median and interquartile ranges. DMSO vs. paxilline **p = 0.0023, DMSO vs. paxilline + tats *p = 0.0133. *p* phosphor, *CREB* cyclic AMP response element binding protein, *DMSO* 0.01% dimethyl sulfoxide control, *tats* scrambled tatCN21 control

As previously demonstrated in whole cells, BK channel inhibition with paxilline increased CREB phosphorylation sevenfold (**p = 0.0023). STO-609 reduced this paxilline-induced CREB phosphorylation by approximately 60% and to a level that was not significantly different to the level of CREB phosphorylation seen in DMSO controls (Fig. 4b). In the tatCN21 experiment, a scrambled peptide linked to the tat sequence (tats) acts as a negative control. Paxilline combined with tats resulted in an approximate fivefold increase in pCREB levels compared to DMSO control (*p = 0.0133). Substitution of tats with tatCN21, the CaMKII inhibitor peptide, reversed the paxilline-induced CREB phosphorylation, resulting in no significant difference between this group and the DMSO control (Fig. 4b). It was also noted that treatment of macrophages with STO-609 or tatCN21 in the absence of paxilline increased CREB phosphorylation by approximately threefold and, twofold respectively, although this was not significant (Fig. 4c).

**Fig. 5:**
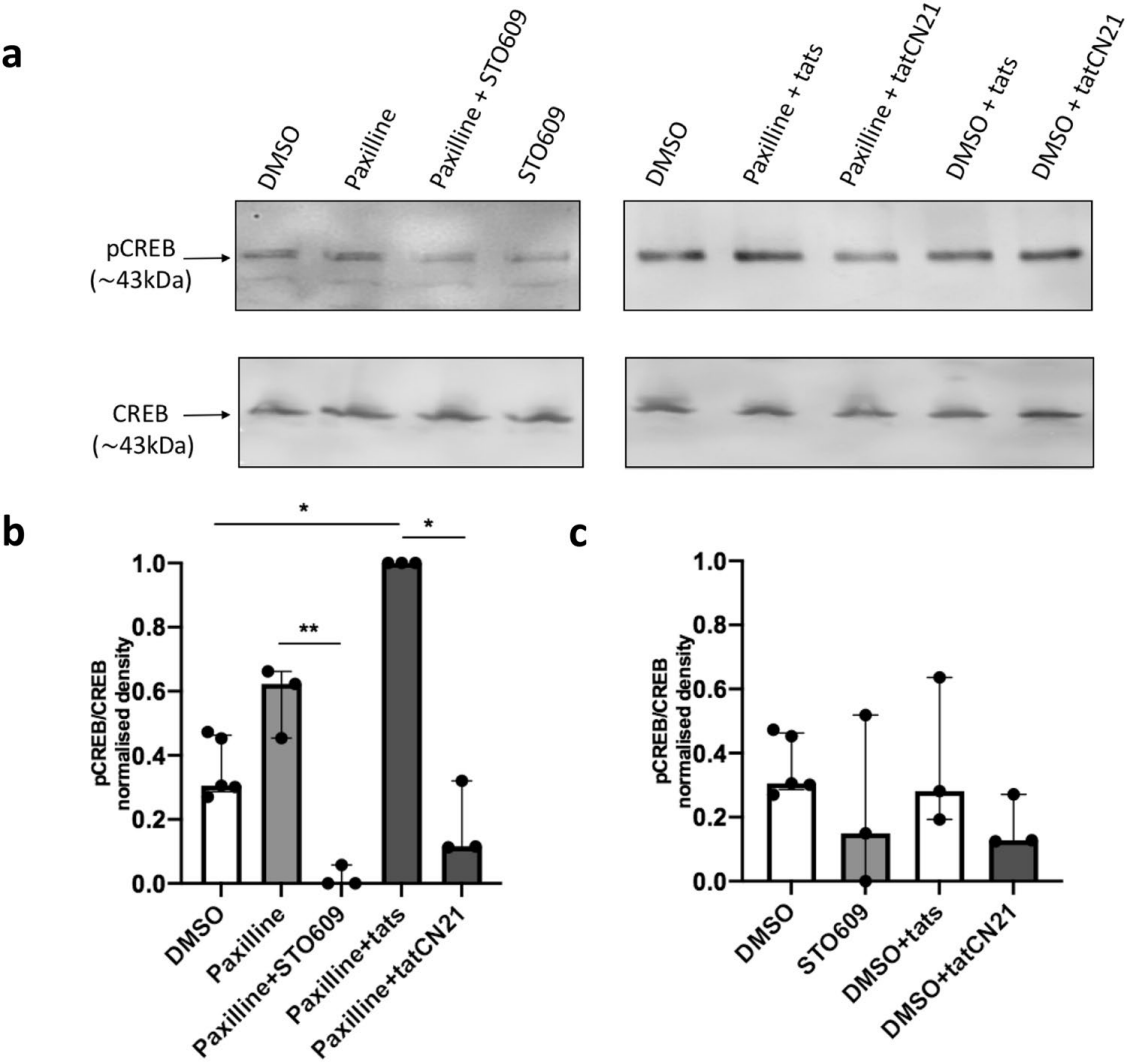
Ca^2+^ and calmodulin-dependent kinase inhibitors reduce paxilline-induced CREB phosphorylation in isolated nuclei. Western blot of pCREB and CREB expression following pre-treatment of isolated nuclei with 5 μM STO609, 5 μM tatCN21, 5 μM tats or 100 mM NaOH for 10 min and subsequent stimulation with 1 μM paxilline or DMSO for 5 min. **a** Data representative of one repeat. Approximate molecular weight of the protein of interest is indicated in kDa. 10 ug protein was loaded into each lane. **b**, **c** Densitometry analysis of immunoblots. The results are representative of at least 3 independent experiments. Kruskal–Wallis one-way analysis of variance, followed by Dunn’s post hoc test. Error bars represent median and interquartile ranges. Paxilline vs. paxilline + STO609 **p = 0.0095, DMSO vs. paxilline + tats *p = 0.0441, paxilline + tats vs. paxilline + tatCN21 *p = 0.0150. *p* phospho, *CREB* cyclic AMP response element binding protein, *DMSO* 0.01% dimethyl sulfoxide control, *tats* scrambled tatCN21 control

CaMKII and CaMKIV can be found in both the cytosol and the nucleus, therefore to confirm a role for the nuclear kinases in CREB phosphorylation associated with BK channel inhibition, we repeated the experiment in isolated nuclei from RAW264.7 macrophages. In paxilline-treated nuclei, CREB phosphorylation was significantly reduced by more than 85% in both the STO609 (**p < 0.0095) and CN21 (*p < 0.015) treated groups compared to the relevant controls, (Fig. 5b). While not significant, it was noted that both STO609 and CN21 reduced CREB phosphorylation in non-paxilline treated controls by approximately 50% (Fig. 5c). Together these results suggest CaMKII and CaMKIV link the BK channel to CREB phosphorylation in the nucleus.

## Discussion

Macrophages are not only central to the inflammatory process but also tissue homeostasis. Since they can produce a wide variety of inflammatory mediators and tissue damaging biochemicals, their activation is tightly regulated [22, 23]. To accomplish this, macrophages have a diverse array of signalling cascades which can respond to changes in the local tissue environment and regulate mediator production which is concordant to the required action. In this paper, we investigated the possibility that nuclear BK channels may have a role in macrophage signalling mechanisms. To the best of our knowledge, we have demonstrated for the first time a role for nuclear located BK channels in regulating macrophage signalling.

Western blot analysis clearly demonstrated the presence of the pore-forming BK channel α-subunit in nuclear and nuclear membrane preparations from RAW264.7 macrophages. In addition to the predicted Western blot band at 120 kDa, numerous studies of BK channel α-subunit expression have described multiple positive bands for the protein, with a doublet band at approximately 120 kDa often being reported. These additional bands most likely represent mRNA splicing or glycosylation events. The absence of standard markers for cytosol, mitochondria and endoplasmic reticulum but the presence of lamin B1 in nuclear preparations would suggest that the presence of the BK channel positive staining in nuclear preparations was not due to contamination of our samples with other organelle material.

CREB is a transcription factor which is associated with a variety of roles in macrophages, particularly the prevention of apoptosis [24, 25]. The cellular function of CREB is dependent on the site of its phosphorylation, with phosphorylation of CREB at Ser133 increasing transcriptional activity of the protein [32]. We demonstrated that 1 μM paxilline, a BK channel blocker, could significantly induce CREB Ser133 phosphorylation to a level similar to that obtained after stimulation of RAW264.7 cells with an optimum concentration of LPS. As the lipid solubility of paxilline suggests no selectivity towards BK channels in different cellular membranes, unless associated with different auxiliary subunits, we investigated the effect of paxilline on isolated nuclei. Paxilline caused an increase in CREB phosphorylation in the nuclei to the same extent as the positive control, 200 nM Ca^2+^ (Fig. 3). While these results do not preclude a role for BK channels located in other cellular membranes in the activation of CREB, they clearly demonstrate that nuclear BK channels are involved in regulating CREB phosphorylation. How nuclear BK channels regulate CREB phosphorylation is not known. However previous work in neurons suggests that blocking BK channels results in an increase in nuclear Ca^2+^ concentration which activates various down-stream calcium sensitive kinases [19]. While calcium regulated signalling pathways have been well documented in macrophages, the specific role of nuclear Ca^2+^ signals in macrophage signalling pathways is not widely studied.

In attempt to elucidate the signalling cascade between BK channels and CREB phosphorylation, we investigated the role of CaMKII and CaMKIV. Both these kinases have been demonstrated to be expressed by macrophages where they have been implicated in a variety of functions [34–36]. Using an inhibitor of CaMKII, CN21, [29] and STO609, a selective inhibitor of the CaMKIV via inhibition of up-stream CaMKK2, [33] we demonstrated that both these kinases are involved in the increased CREB phosphorylation seen after BK channel inhibition with paxilline treatment (Figs. 4 and 5). Importantly, it was demonstrated that the CREB phosphorylation associated with BK channel inhibition was reduced in isolated nuclei with inhibitors of CaMKs. This suggest that the CaMKs are downstream of the BK channel in the nucleus. It was also noted that in resting whole cells, i.e. in the absence BK channel inhibition with paxilline, inhibition of these kinases caused an apparent increase in CREB phosphorylation. In comparison, in isolated nuclei, which were not pre-treated with paxilline, CaMK inhibition caused a decrease in CREB phosphorylation. While these results are not significant in our experiments, these data may relate to differing roles that cytosolic and nuclear kinases play in CREB phosphorylation in macrophages under various conditions.

Only relatively few studies have investigated CaMKs in macrophages [34–36]. In a recent study, CaMKII inhibition led to the generation of M1 type macrophages [37], a classically activated pro-inflammatory macrophage phenotype which produces pro-inflammatory mediators and has anti-tumours activity. However, in a second study, suppression of CaMKII activity in macrophages was linked to inflammation resolution which is typically associated with an anti-inflammatory macrophage phenotype [38]. While these reports appear contradictory, the contribution of different CaMKII isoforms, and importantly cellular location of the kinases was not investigated, and this may be an important factor to understanding the effects of CaMKs in macrophage function. Future work will investigate the role of Ca^2+^ and cytosolic/nuclei located CaMKs in macrophage function [32].

To our knowledge, this is the first report demonstrating a functional role of BK channels in macrophage CREB activation, or indeed of any nuclear ion channels in a macrophage signalling cascade. Several important questions need to be addressed such as how do cytoplasmic signalling pathways interact with nuclear BK channels? What are the nuclear signalling pathways modulated by nuclear BK channels? And how does nuclear BK channel signalling regulate macrophage cellular functions? However, this paper suggests that the roles of nuclear channels, and in particular BK channels, in macrophage function should be further investigated.

## Supplementary Information

Below is the link to the electronic supplementary material.

Supplementary file1 (DOCX 32180 KB)

## Abbreviations

BK channel: Large conductance voltage- and Ca^2^^+^-activated potassium channel
nBK: Nuclear BK channel
pmBK: Plasma membrane BK channel
cAMP: 3′,5′-Cyclic adenosine monophosphate
CaMK: Ca^2^^+^ and calmodulin-dependent protein kinase
CaMKII: Ca^2^^+^ and calmodulin-dependent protein kinase type-II
CaMKIV: Ca^2^^+^ and calmodulin-dependent protein kinase type-IV
CaMKK2: Ca^2^^+^ and calmodulin-dependent kinase kinase 2
CREB: CAMP Response Element Binding Protein
COX-2: Inducible cyclooxygenase-2
DTT: Dithiothreitol
FBS: Fetal bovine serum
HRP: Horse radish peroxidase
LPS: Lipopolysaccharide
NF-κB: Nuclear factor-kappa B
PRRs: Pattern recognition receptors (PPRs)
TLR-4: Toll-like receptor-4
TNFα: Tumour Necrosis Factor-alpha
