# Supplementary material for: Nuclear BK channels regulate CREB phosphorylation in RAW264.7 macrophages
Source: Pharmacol Rep. 2021 Mar 13;73(3):881–90. doi: 10.1007/s43440-021-00229-z (PMC8180476; doi:10.1007/s43440-021-00229-z)
Supplement: Supplementary file 1 — Supplementary file1 (DOCX 32180 KB) [file 43440_2021_229_MOESM1_ESM.docx]

**Supplemental material.**

Original images.

Figure 1a

Lamin B1, loaded for cell No. BK Cannel, loaded for cell No.


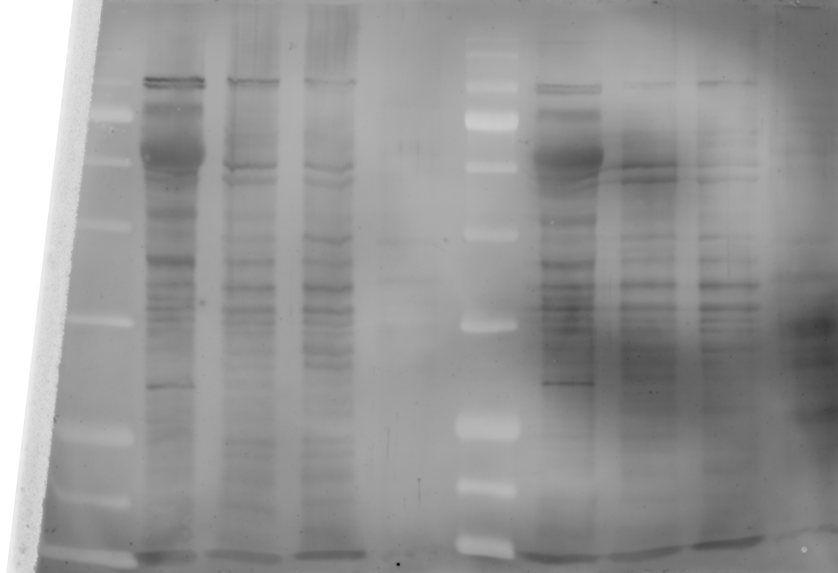


80

58

46

32

110


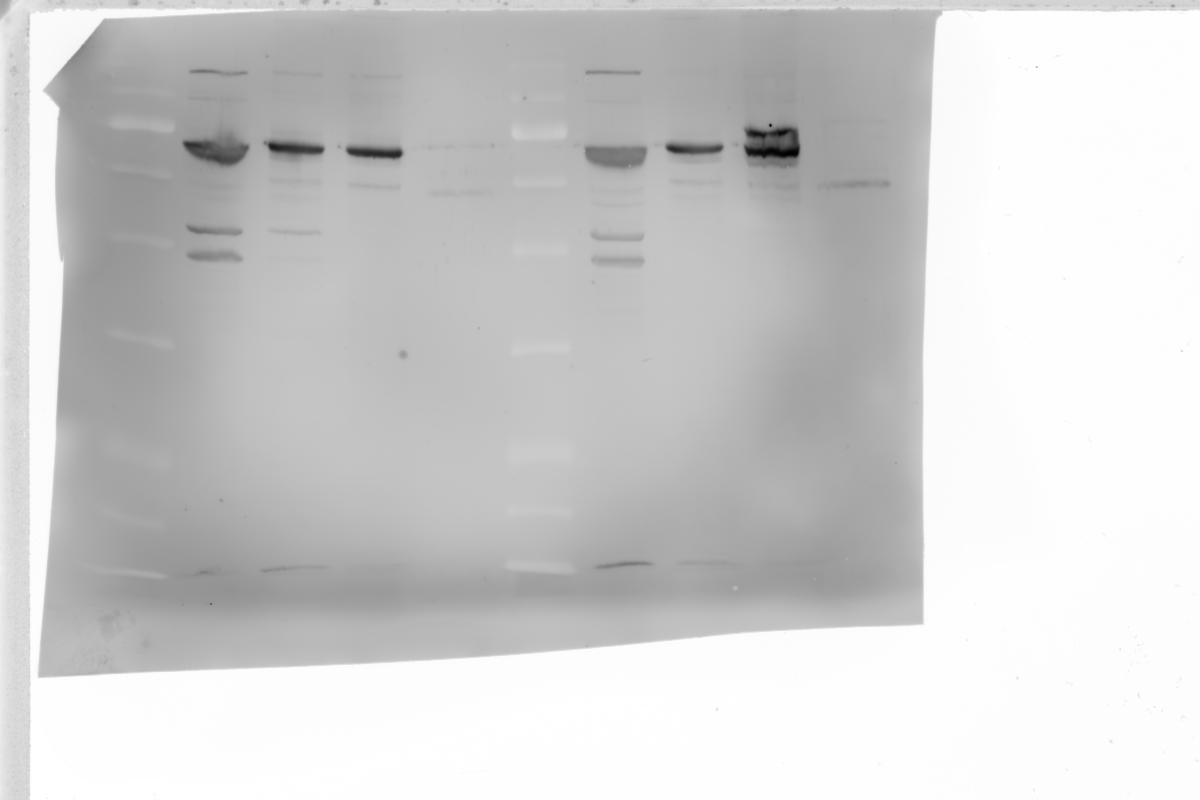


80

58

46

32

80

58

46

32

110


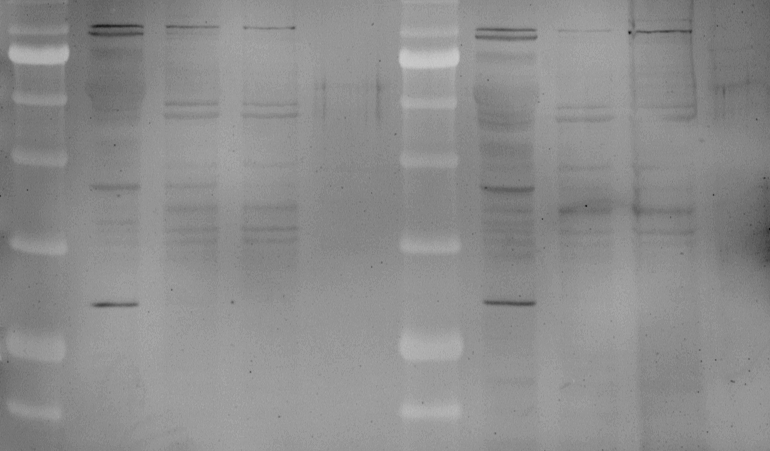

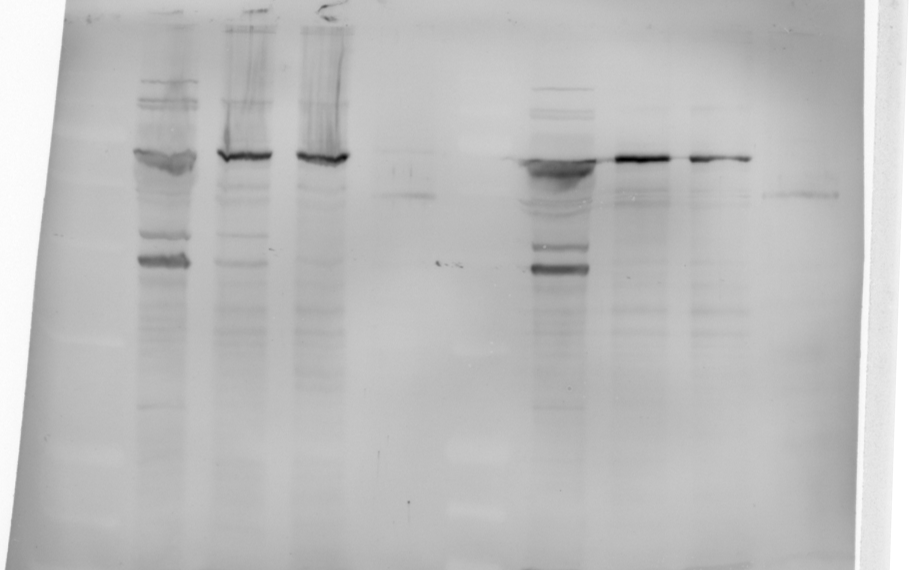


80

58

46

32

Lamin B1, loaded for protein. BK Cannel, loaded for protein.

Figure 1b


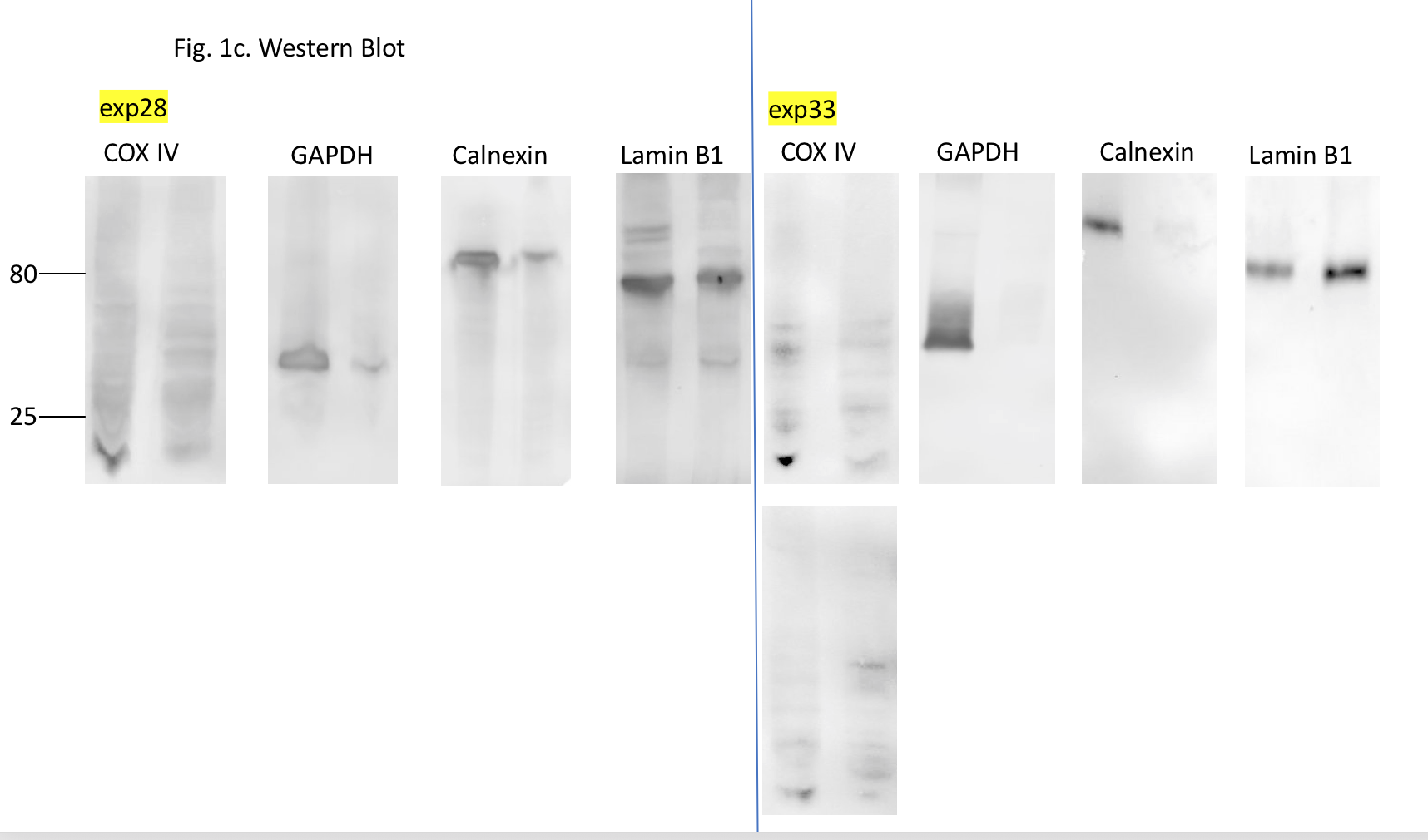

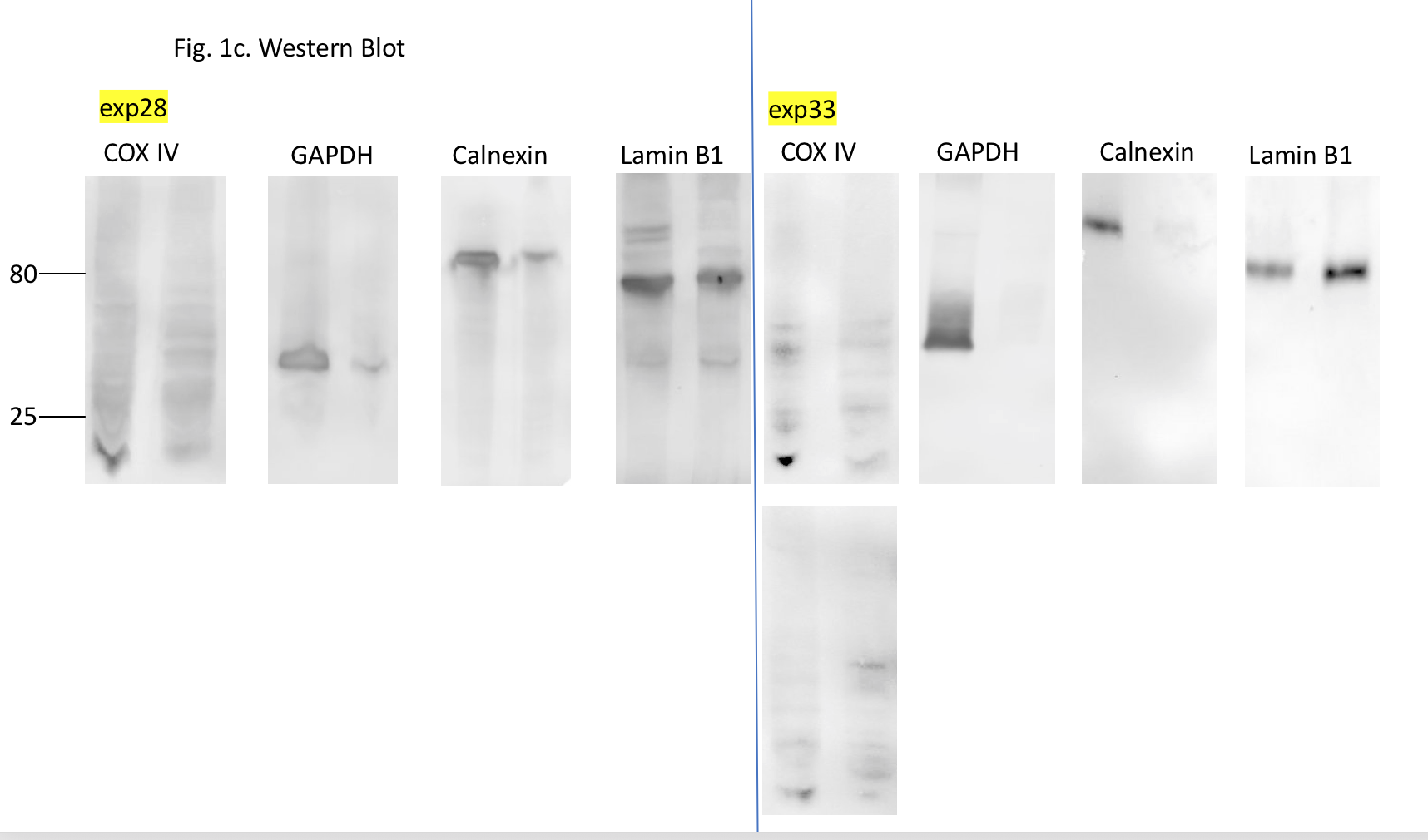

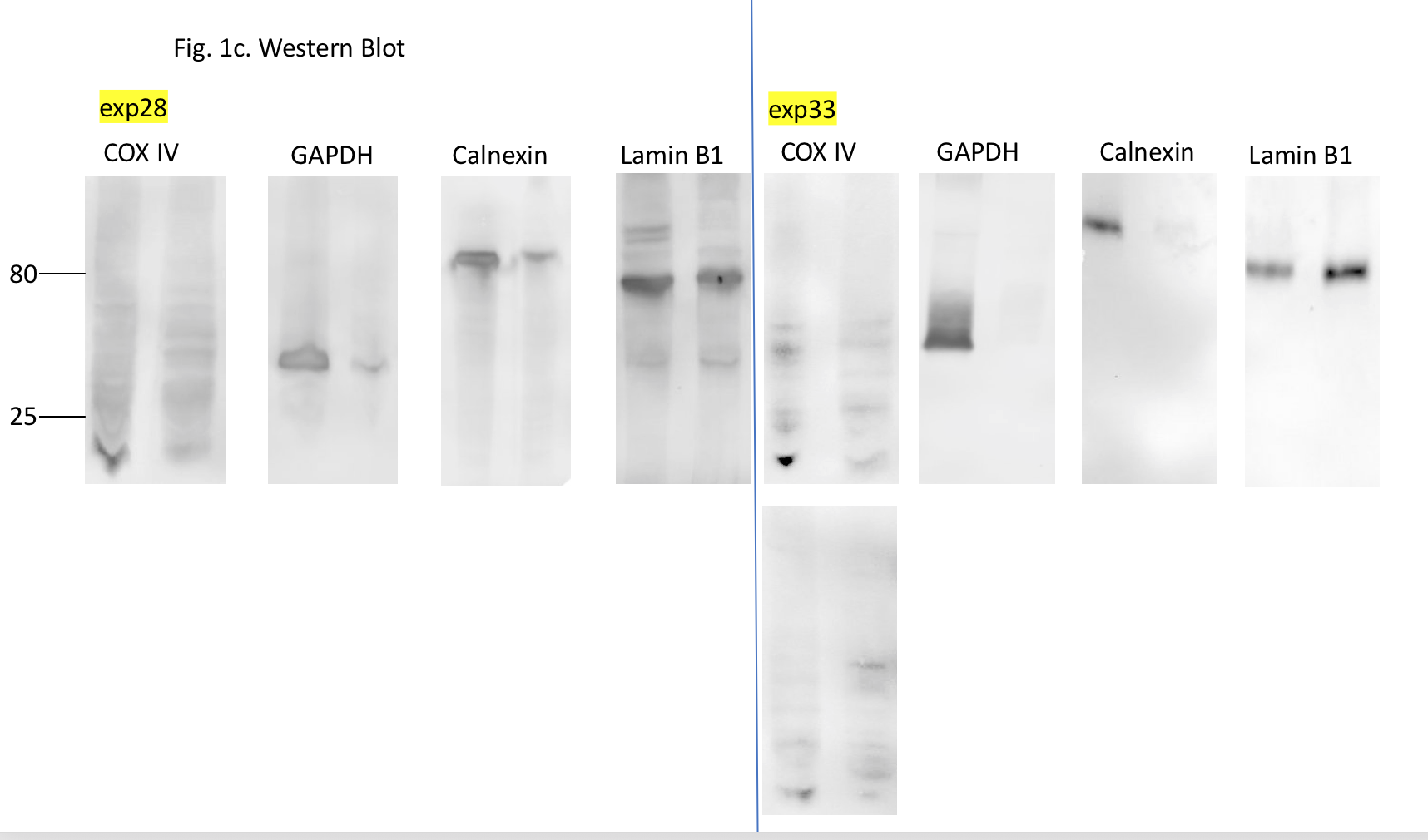

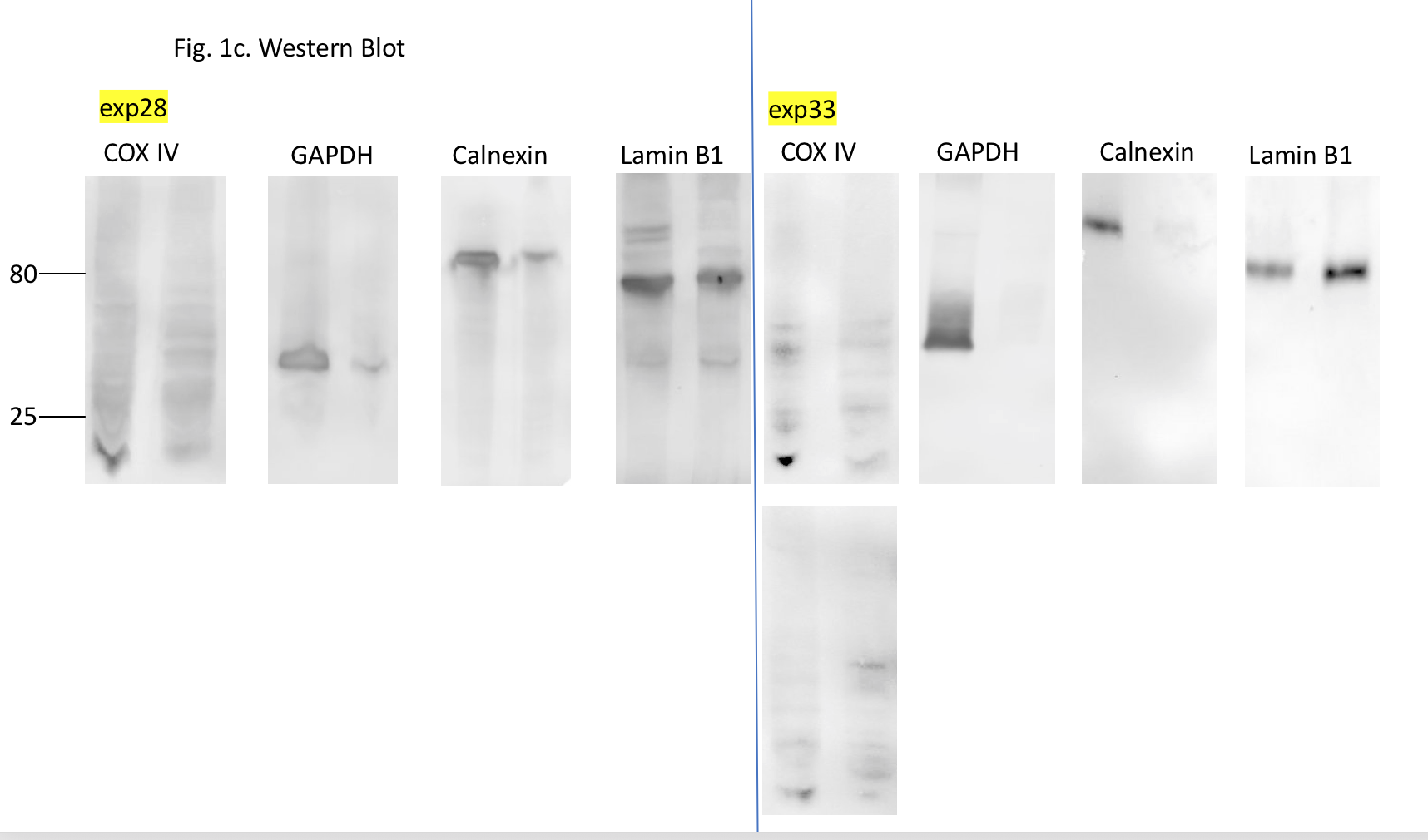


80

25

80

25

Lamin B1

COX IV

80

25

GAPDH

Calnexin

80

25

Figure 1c


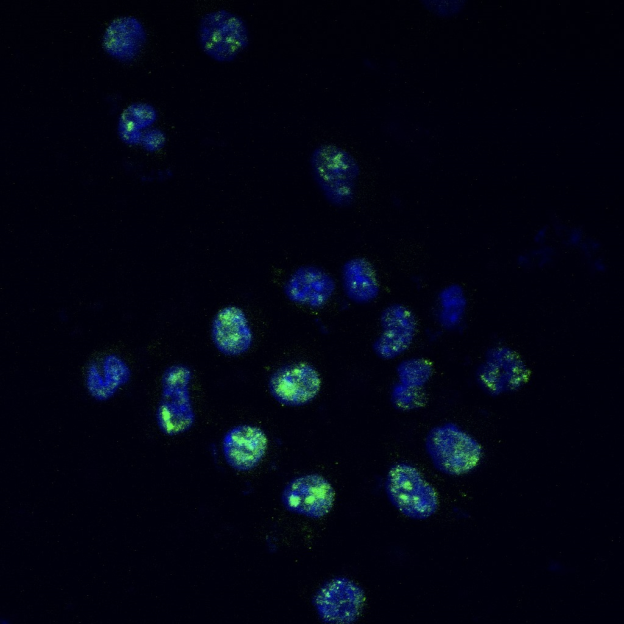

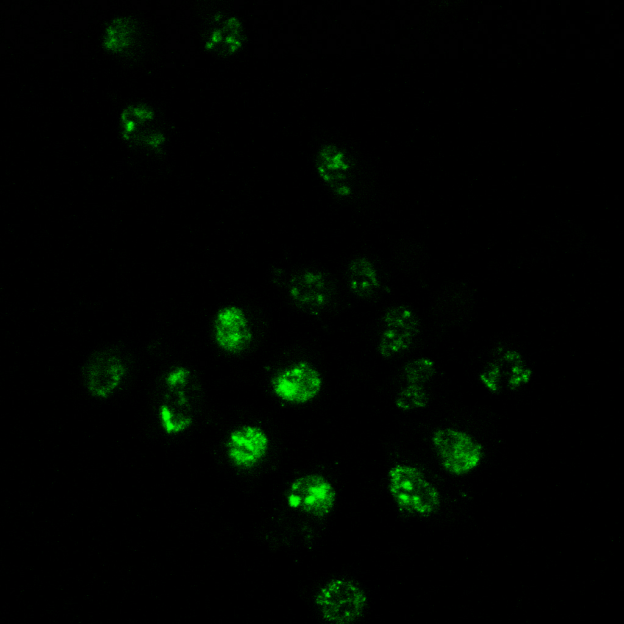

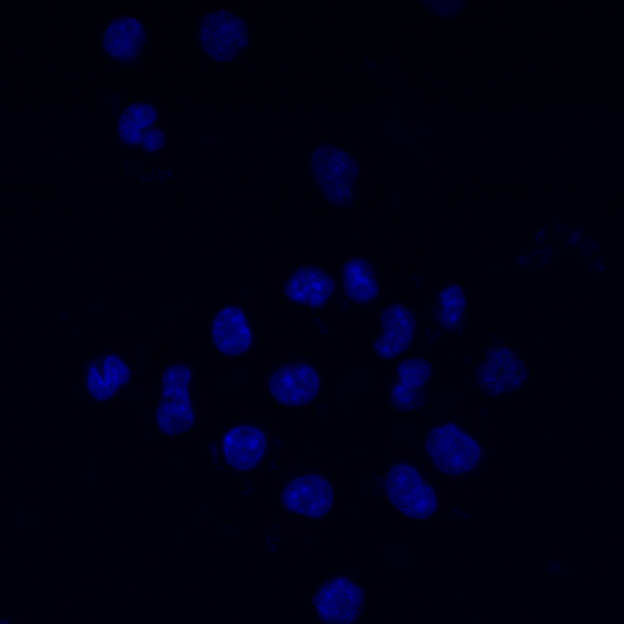


Figure 2

CREB


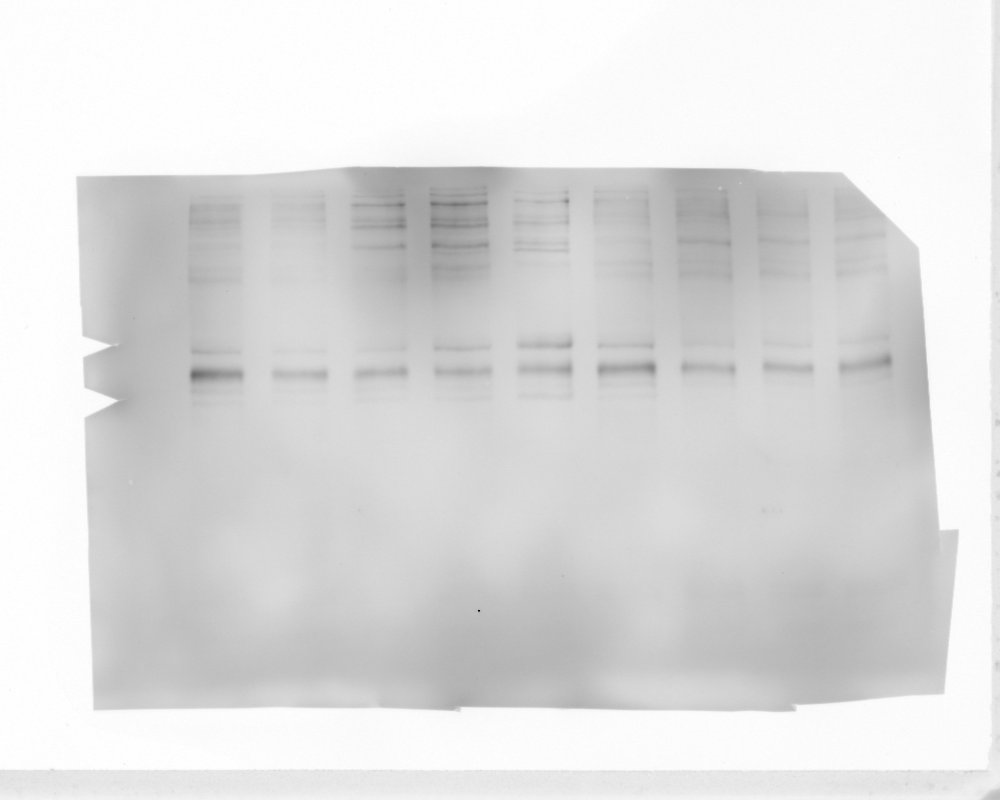


35

58

pCREB


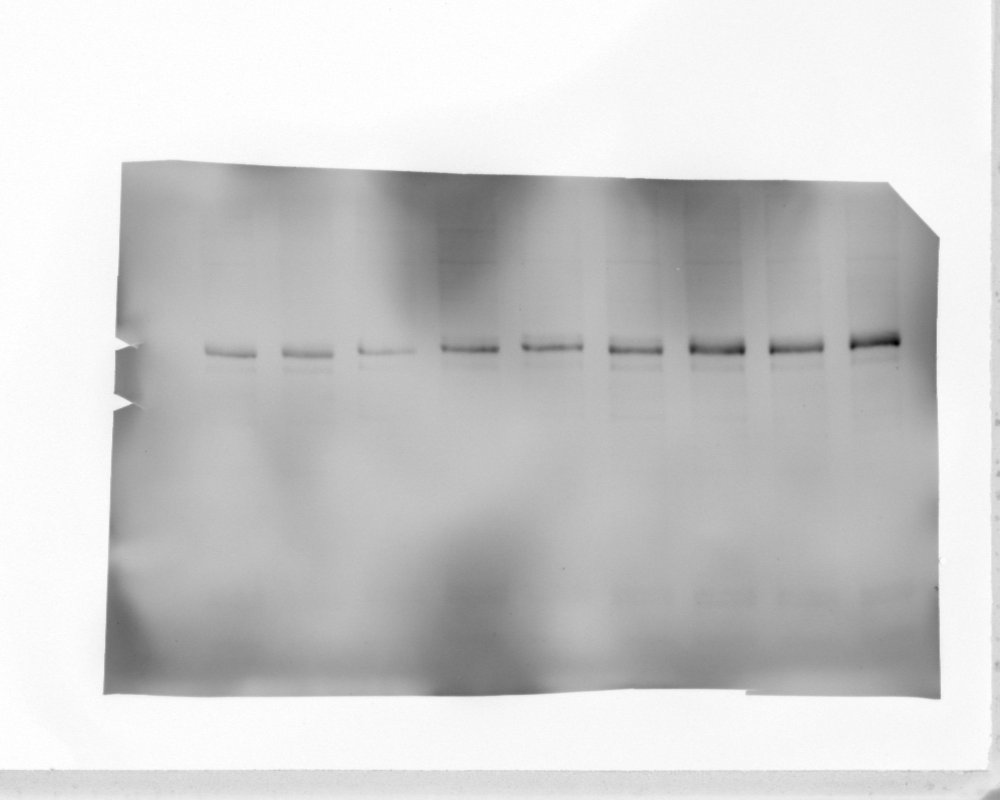


35

58

Figure 3

CREB


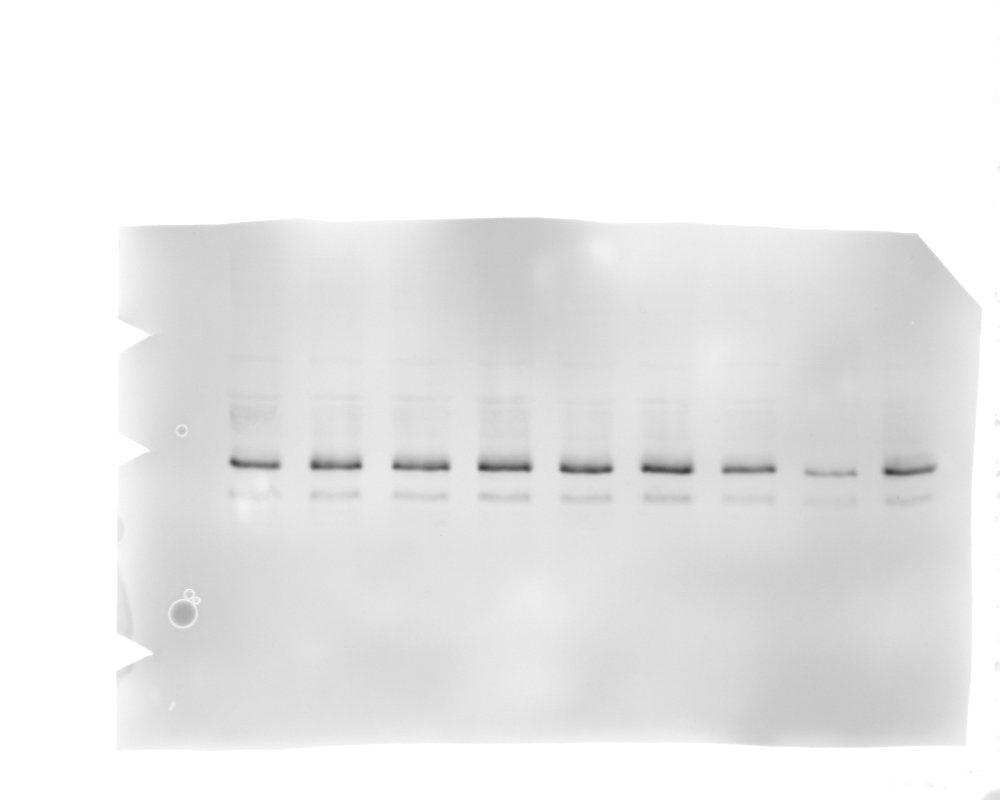


58

35

pCREB


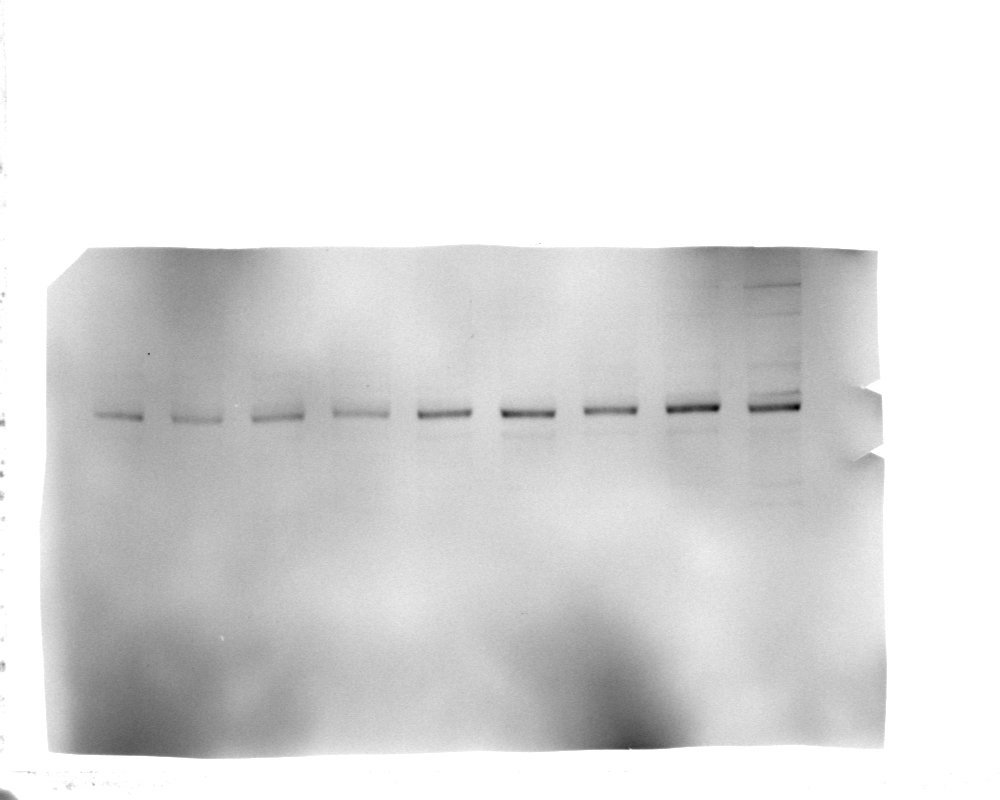


58

35

Figure 4

CREB (left) CREB (right)


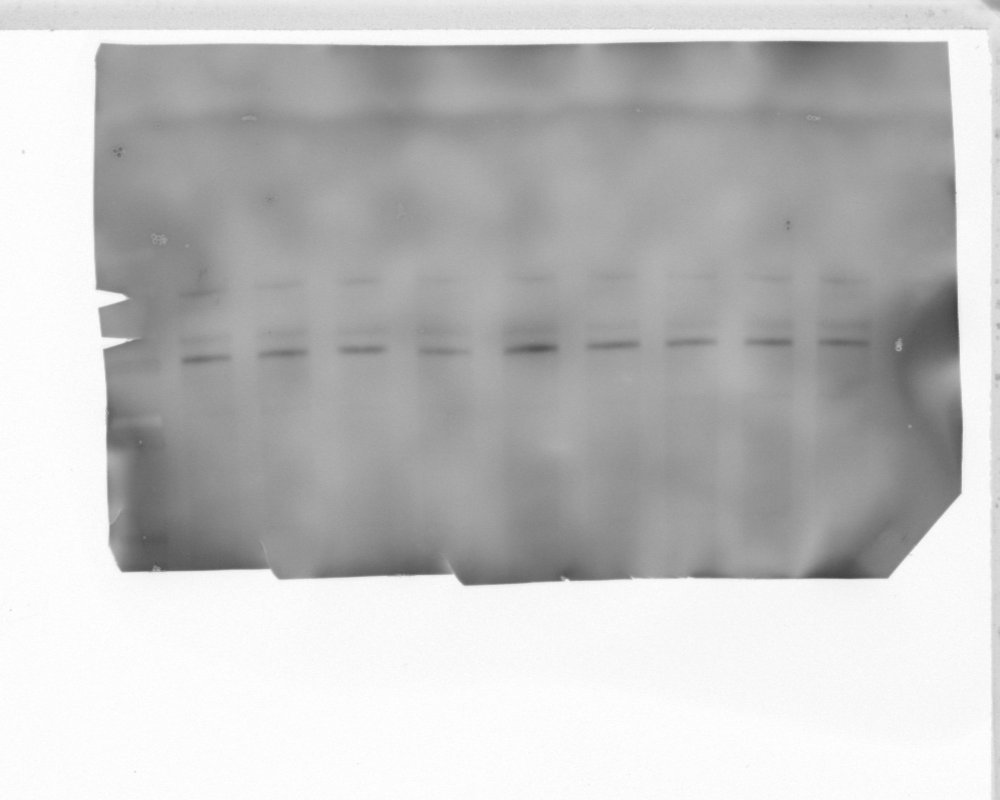


58

35


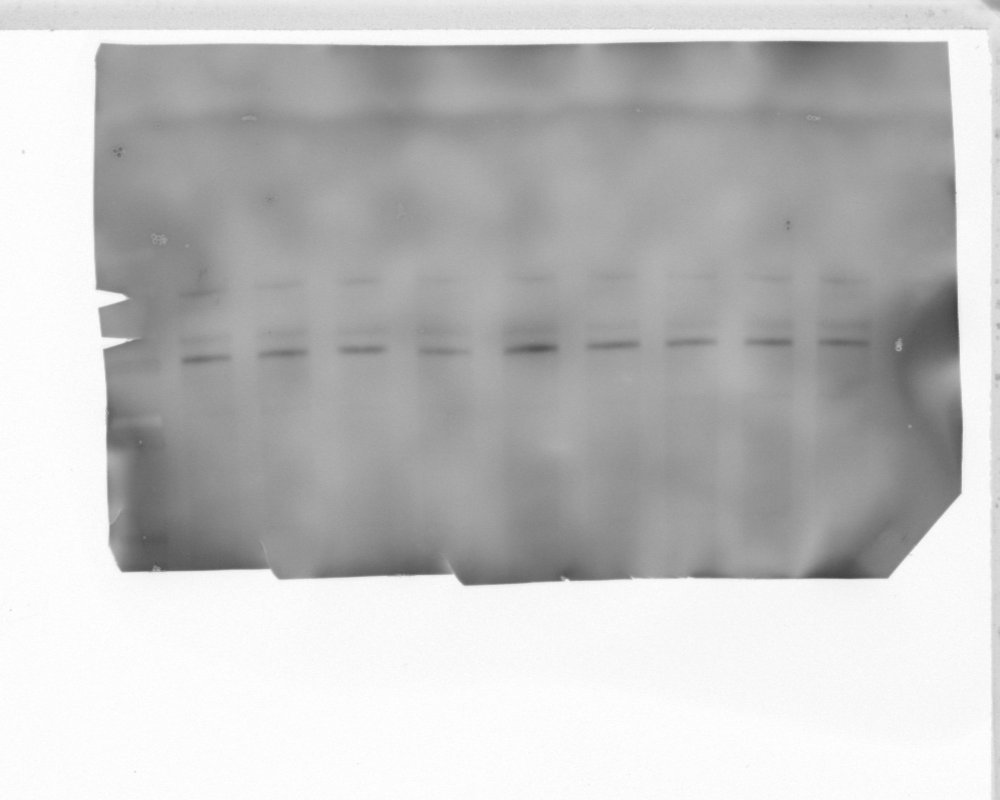


58

35

pCREB (left) pCREB (right)


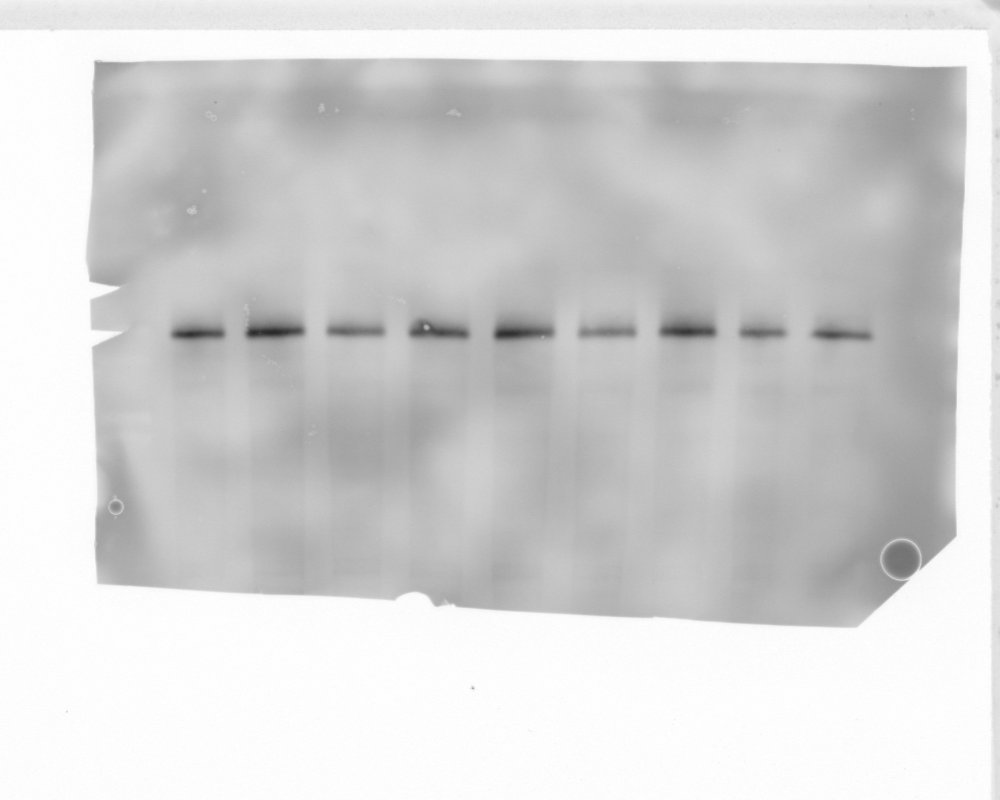


35

58


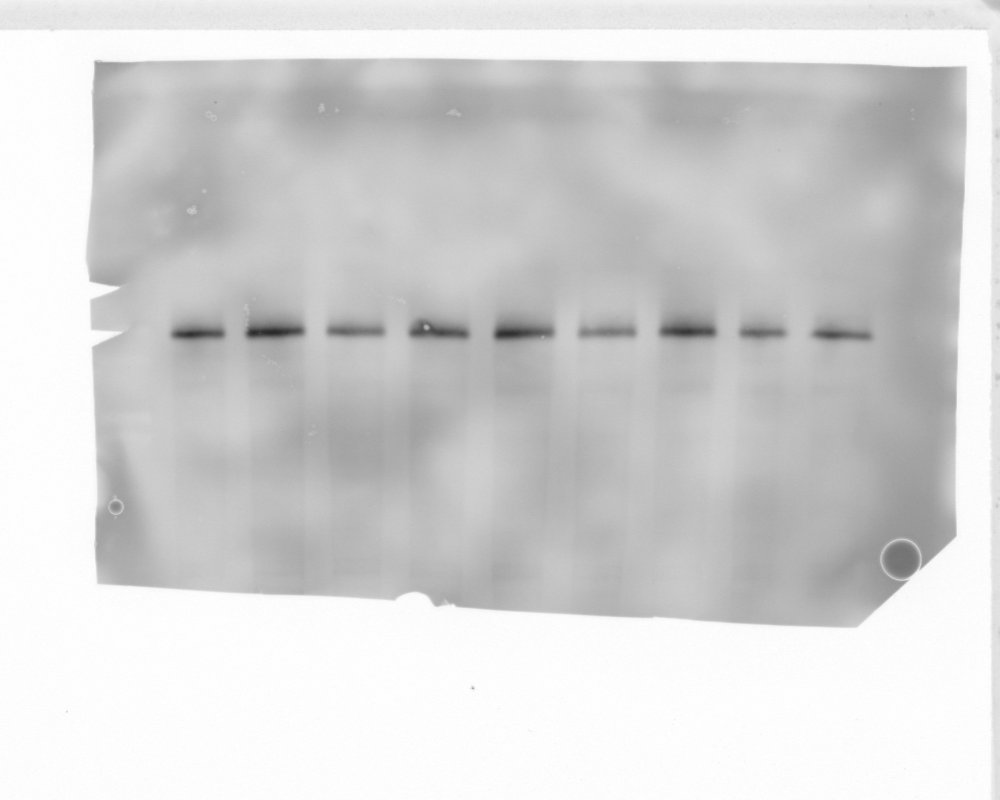


58

35

Figure 5

CREB (left) CREB (right)


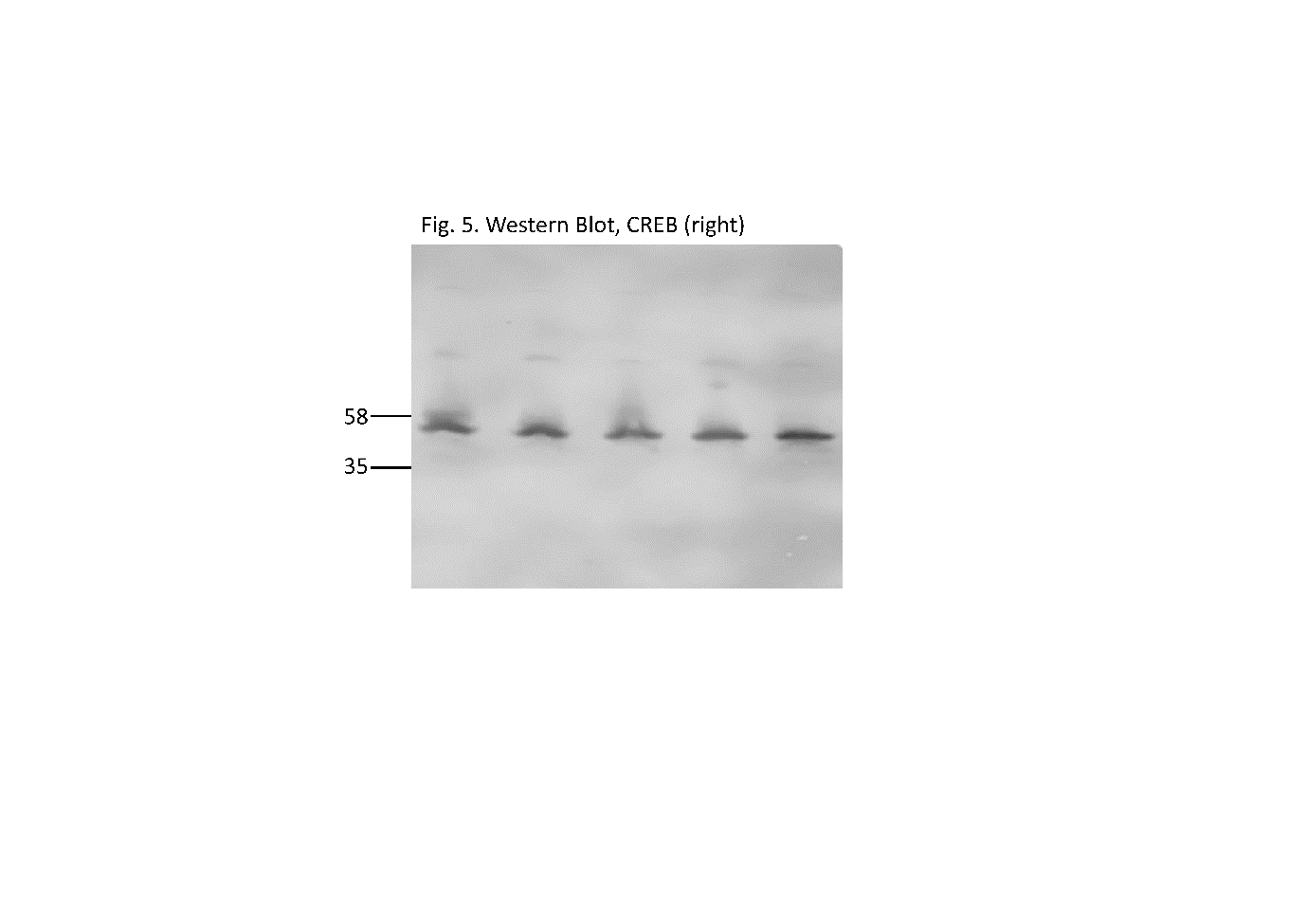


35

58


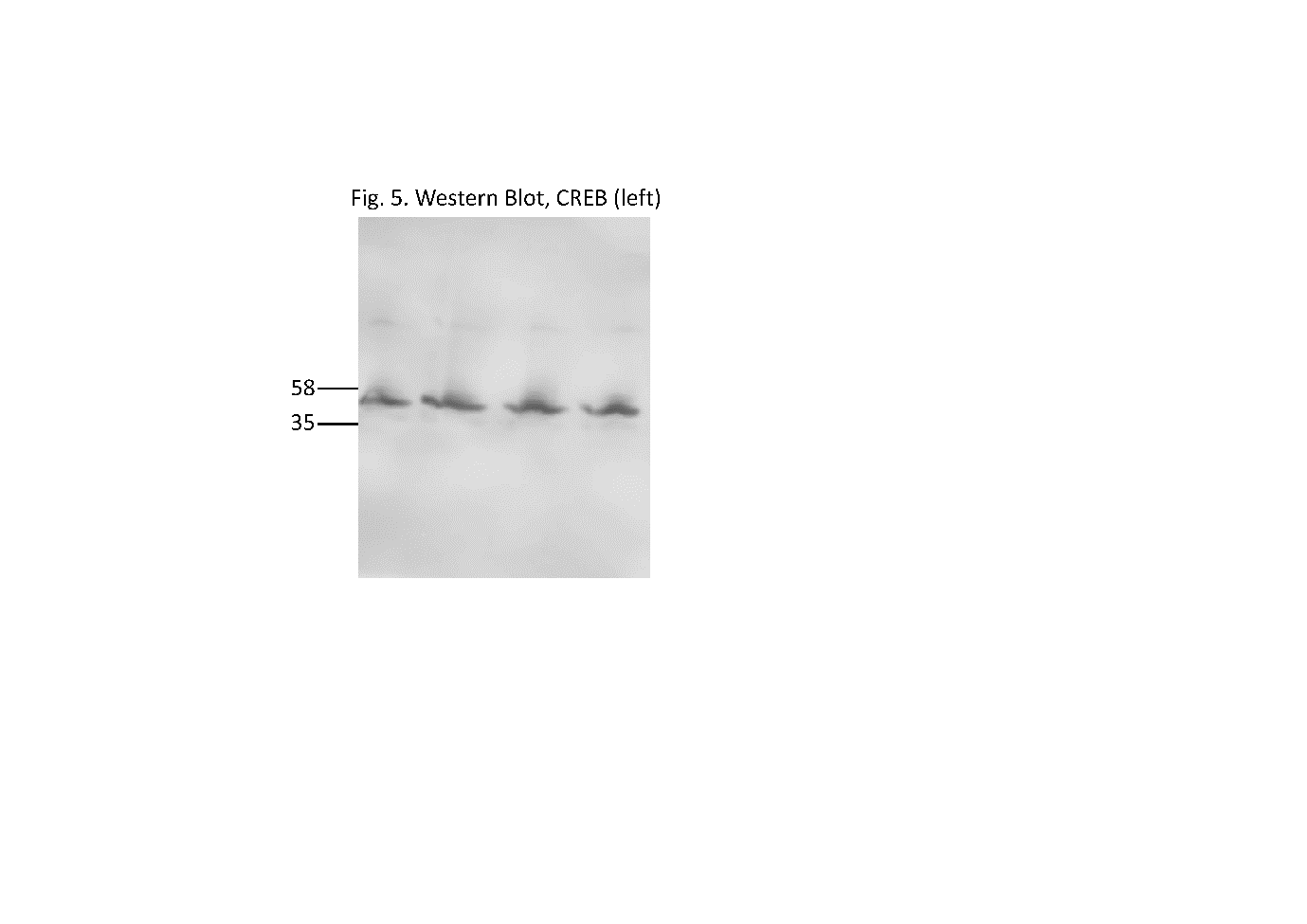


35

58

pCREB (left) pCREB (right)

35

58


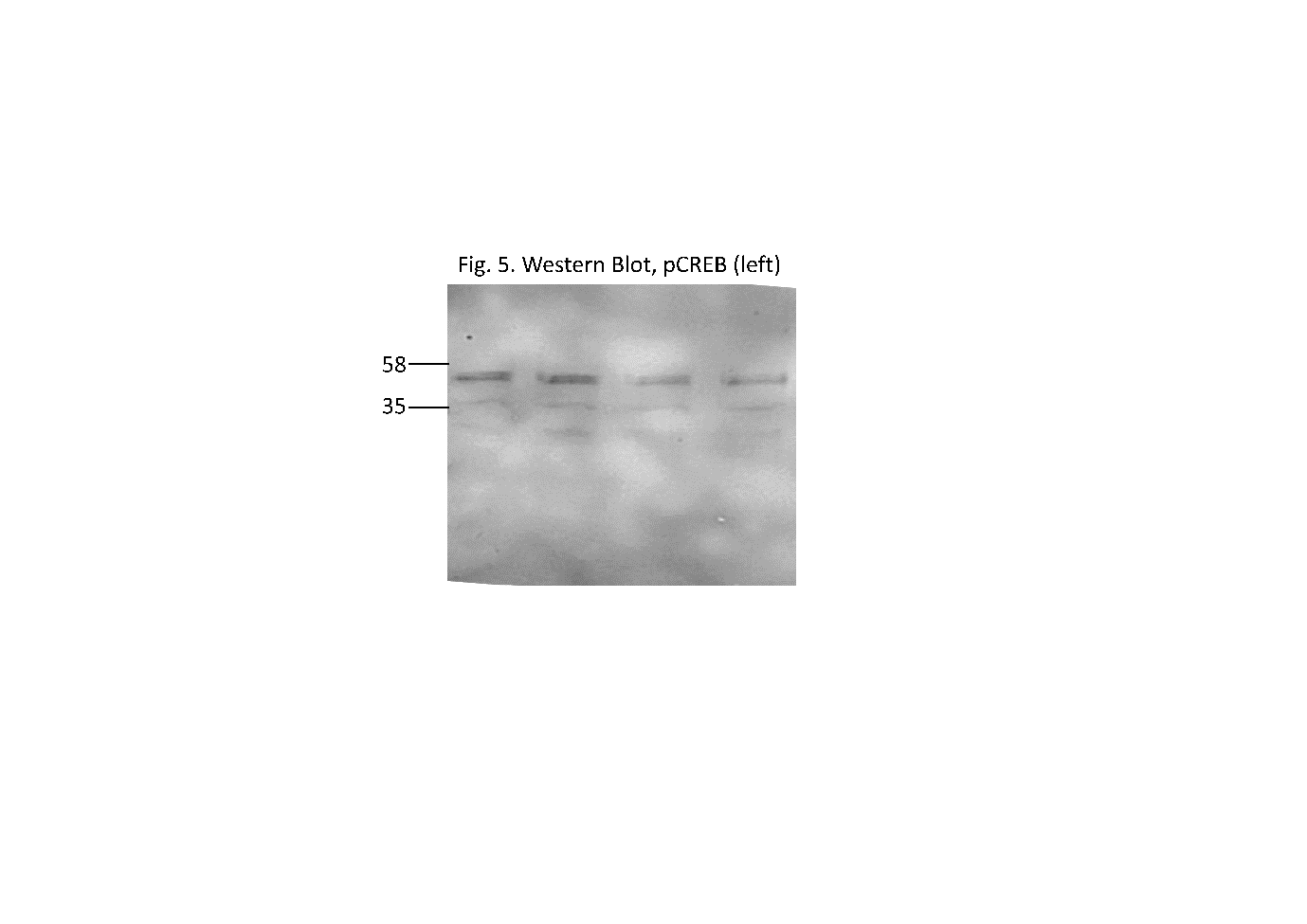

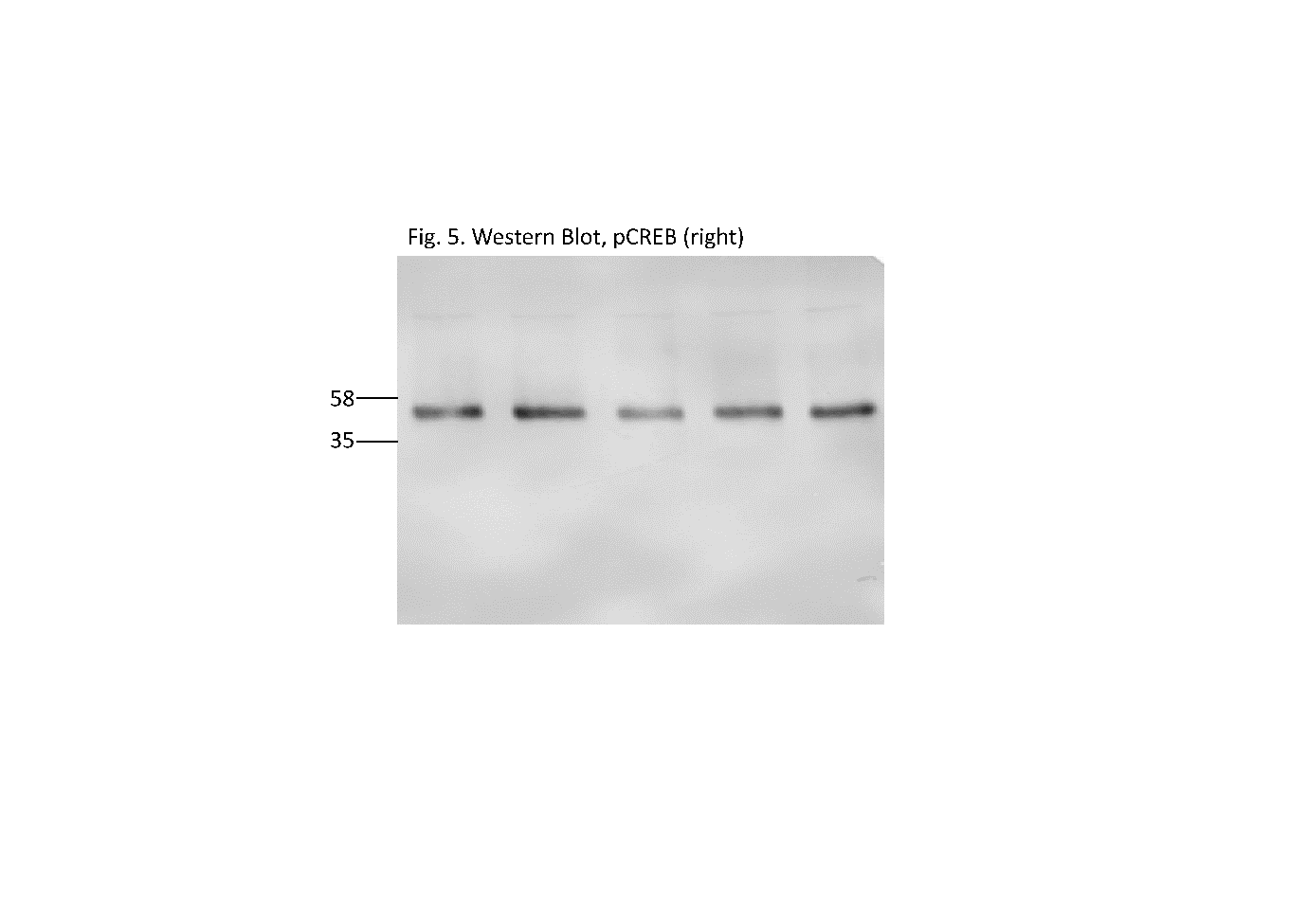


35

58

Supplementary Figure 1

**a**

LPS (ng/ml)

TNFα (ng/ml)

ND

**b**

Con

DMSO

30

100

300

1000

3000

0

250

500

750

1000

5

10

15

20

25

0

TNFα (pg/ml)

ND

ND

ND

ND

ND

ND

3

10

30

100

300

1000

1

0

Paxilline nM

**Supplementary Figure 1. Effect of LPS and paxilline on TNFα release from macrophages.** Cells were stimulated with the indicated drugs for 4 hours and medium collected. TNFα concentration in cell medium was measured by ELSIA. a) LPS dose response 1-1000ng/ml. b) Paxilline dose response 30-3000nM. The results are from 4 independent experiments. Limit of detection is 142pg/ml TNFα based on replicate blank measurements + 3 S.D. Bars represent mean ± S.E.M. ND-Not detected; Con-medium only; DMSO- 0.01% DMSO.

Statistical analysis Report.

| Figure number | Normality test | Passed normality test? | Test | Descriptive statistics | H value | Sample size | P value | Post hoc test | P values |
| --- | --- | --- | --- | --- | --- | --- | --- | --- | --- |
| **2b** | Shapiro-Wilk test | NO | Kruskal-Wallis test | Median with interquartile range | 14.97 | N_1_=7, N_2_=6, N_3_=8, N_4_=5,  N_5_=7, N_6_=5 | 0.0105 | Dunn’s test | P _(NT vs. 100ng/ml LPS)_  =0.0034  P _(DMSO vs. 1000nM Paxilline)_  =0.0132 |
| **3b** | Shapiro-Wilk test | NO | Kruskal-Wallis test | Median with interquartile range | 11.67 | N_1,2,3,4,5_=3 | 0.0012 | Dunn’s test | P _(NT vs. 200nM Ca_^2+^_)_  =0.0168  P _(DMSO vs. 100nM Paxilline)_  =0.0168 |
| **4b** | Shapiro-Wilk test | YES | Kruskal-Wallis test | Median with interquartile range | 12.86 | N_1_=8, N_2,3,4,5_=4 | 0.0120 | Dunn’s test | P _(DMSO vs. Paxilline)_  =0.0023  P _(DMSO vs. Paxilline+tats)_  =0.0133 |
|  |  |  | Ordinary one-way ANOVA | n/a | F =  6.451 |  | 0.0019 | Fisher’s LSD test | P _(DMSO vs. Paxilline)_  =0.0003  P _(Paxilline vs. Paxilline+STO609)_  =0.0432  P _(DMSO vs. Paxilline+tats)_  =0.0048  P _(Paxilline+tats vs. Paxilline+tatCN21)_  =0.0268 |
| **4c** | Shapiro-Wilk test | NO | Kruskal-Wallis test | Median with interquartile range | 4.539 | N_1_=8, N_2,3,4,5_=4 | 0.2089 | n/a | n/a |
| **5b** | Shapiro-Wilk test | NO | Kruskal-Wallis test | Median with interquartile range | 14.29 | N_1_=5, N_2,3,4,5_=3 | 0.0064 | Dunn’s test | P _(Paxilline vs. Paxilline+STO609)_  =0.0095  P _(DMSO vs. Paxilline+tats)_  =0.0441  P _(Paxilline+tats vs. Paxilline+tatCN21)_  =0.0150 |
| **5c** | Shapiro-Wilk test | NO | Kruskal-Wallis test | Median with interquartile range | 4.131 | N_1_=5, N_2,3,4_=3 | 0.2654 | n/a | n/a |
